# Heatwaves Constrain the Future Persistence of Mosquito Vectors in Europe

**DOI:** 10.1111/gcb.70876

**Published:** 2026-04-27

**Authors:** Isabelle Marie Kramer, Stien Vereecken, Adwine Vanslembrouck, Younes Smekens, Jacobus de Witte, Sofia Vielma, Aidin Niamir, Ruth Müller

**Affiliations:** ^1^ The Unit of Entomology, Department of Biomedical Sciences Institute of Tropical Medicine Antwerp Belgium; ^2^ Institute of Occupational, Social and Environmental Medicine Goethe University Frankfurt Frankfurt Germany; ^3^ Senckenberg Biodiversity and Climate Research Centre Frankfurt am Main Germany; ^4^ Department of Biomedical Sciences, Faculty of Pharmaceutical, Biomedical and Veterinary Sciences University of Antwerp Antwerp Belgium

**Keywords:** extreme heat, range limits, range shifts, temperature tolerance, thermal adaptation, thermal limits

## Abstract

Climate warming is intensifying heatwaves across Europe, but the upper thermal limits of arboviral mosquito vectors—and their implications for future distribution—remain poorly understood. Using experimentally derived life‐stage‐specific upper thermal limits, we identified the impact of heat extremes on the persistence of *Culex pipiens* , *Aedes albopictus* and *Aedes aegypti* in Europe under present and future climate scenarios (SSP126, SSP370 and SSP585). Our projections reveal that by 2100, large parts of southern Europe, including the Iberian Peninsula, will exceed the thermal limits for *Cx. pipiens*, with heat‐limited zones expanding northward into central Europe. *Aedes albopictus* faces moderate future constraints, while *Ae. aegypti* remains largely unrestricted by extreme heat, though high mortality at low humidity may still limit its establishment in continental Europe. Divergent plasticity in heat tolerance among life‐stages, species, their acclimation status and the interplay with humidity exposure underscore the complexity of thermal adaptation. This integrative experimental‐modelling framework highlights when and where it may become too hot for Europe's major mosquito vectors, refining spatial risk forecasts for arbovirus emergence under climate change.

## Graphical abstract

Climate warming is intensifying heatwaves across Europe, with the potential to constrain the future distribution of major arboviral mosquito vectors. Using life‐stage‐specific upper thermal limits and an integrative experimental‐modelling framework, we show that extreme heat may reduce the persistence of *Culex pipiens* and, to a lesser extent, *Aedes albopictus* in southern and central Europe, while *Aedes aegypti* , when established, remains comparatively heat tolerant under future climate scenarios. The graphical abstract was created in BioRender. Kramer, I. (2026) https://BioRender.com/7ec6cn7.

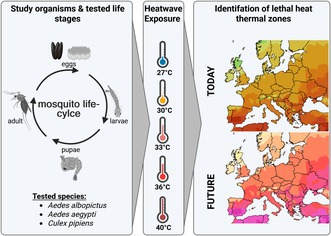

## 1 Introduction

**FIGURE 1:**
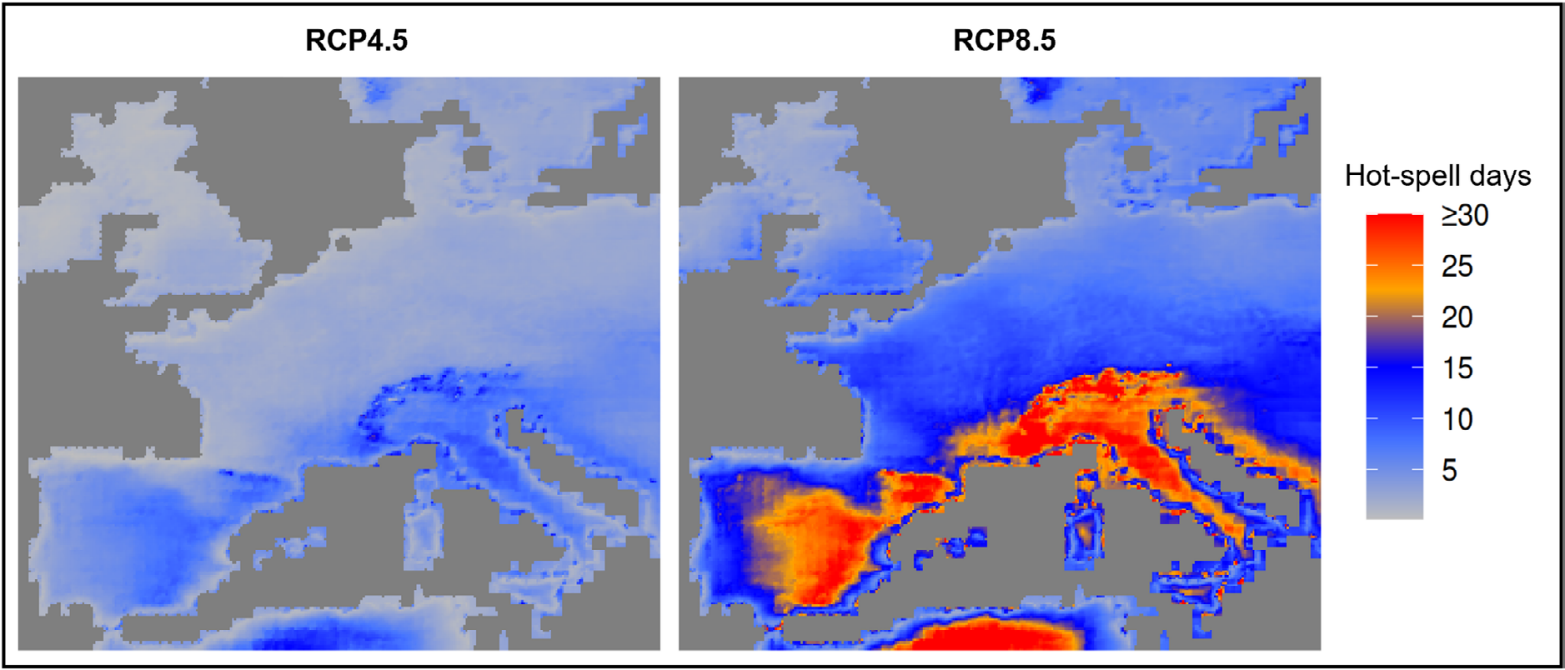
Increase of consecutive days of extreme heat over Europe under climate change scenarios. Hot Spell Days (HSD) are derived from the Copernicus Climate Change Service (C3S) Heat and Cold Spells indicator, defined as calendar days within a heatwave event (≥ 3 consecutive days with daily maximum temperature exceeding a percentile‐based threshold from a historical reference period). Data are provided as annual counts on a 0.1° grid and as bias‐adjusted multi‐model projections under RCP4.5 and RCP8.5. Shown are mean HSD for 1991–2020 and 2071–2100, and the difference between these periods (Copernicus Climate Change Service [C3S] 2019); Hooyberghs et al. 2019.

Driven by climate warming, global temperatures are increasing, and heat events, including heatwaves defined as periods of up to five consecutive hot days, are projected to become increasingly frequent and intense (Hong et al. 2022; IPCC 2022). These short‐term extreme heat events are expected to exert significant ecological pressure on ectothermic organisms, such as mosquitoes, by pushing them closer to their upper thermal limits (UTL). In turn, this may reshape the risk of mosquito‐borne disease transmission and the patterns of respective arboviral vector distribution. So far in 2025, France has reported six outbreaks of locally acquired cases of chikungunya fever, with symptom onset in late May or June. This indicates a very early start of the mosquito season. In previous years, such cases occurred in July or August, highlighting how changing environmental conditions are creating longer and more favourable periods for transmission (Farooq et al. 2025). Driven by climate warming, arboviral vector species must withstand higher temperatures, especially in Europe—the fastest‐warming continent—where temperatures have risen at about twice the global rate since the 1980s (EEA 2024). Not only rising mean temperatures, but particularly increasingly frequent heatwaves pose potentially a challenge to the mosquito's survival (Couper, Nalukwago, et al. 2024; Figure 1). It is uncertain for how long medically relevant mosquito species can tolerate extreme heat, how they adapt physiologically or behaviourally, react through shifts in their distribution or seasonal and/or diurnal activity patterns, and ultimately decline due to heat stress (Couper et al. 2021; Couper, Farner, et al. 2024; Waldvogel et al. 2020). In addition to heat‐induced effects on mosquito physiology, elevated heat levels can either reduce the risk of arboviral infections by surpassing UTLs for transmission or, on the contrary, increase transmission potential by accelerating viral replication and shortening the extrinsic incubation period (Mourya et al. 2004; Sim et al. 2007; Yadav et al. 2005; Zhao et al. 2010).

To assess mosquito survival under heat stress, researchers employ various experimental approaches. These include: (a) life‐cycle experiments involving prolonged temperature exposure to extreme temperatures from the egg or L1 larval stage until death (Delatte et al. 2009; Eisen et al. 2014; Kramer et al. 2021; Moser et al. 2023); and (b) heat shock experiments, in which mosquitoes are subjected to short‐term exposure to high temperatures over several hours—so‐called thermal knockdown experiments—to identify critical thermal maximum (CT_max_), the temperature at which coordinated movement is lost (Andersen et al. 2006; Chakraborty et al. 2025; Diamond and Yilmaz 2018; Richardson et al. 2011; Ware‐Gilmore et al. 2023). Next to survival, behavioural changes in response to high temperatures—collectively referred to as behavioural thermoregulation—are also regularly investigated. These include (c) thermal preference assays to determine preferred temperature ranges (Verhulst et al. 2020), as well as (d) assays examining the impact of heat on blood‐feeding behaviour (Costanzo and Occhino 2023). Just recently, experimental heatwave‐like approaches were applied to *Ae. albopictus* and *Ae. aegypti* life stages to investigate life‐stage‐specific responses to heatwaves for the first time (Alfaro et al. 2026; Covey et al. 2025; Dobbs et al. 2026).

Current evidence suggests that the invasive species *Aedes aegypti* exhibits the highest survival under heat stress, followed by the invasive species *Aedes albopictus* , while the more cold‐adapted native *Culex pipiens* shows the lowest UTL (Kramer 2021; Moser et al. 2023). In heat shock experiments, temperatures above 40°C were regularly tested, while in life‐cycle experiments, temperatures between 33°C and 40°C were examined (Gray 2013; Kramer 2021; Moser et al. 2023; Orlinick et al. 2023; Richardson et al. 2011). In *Aedes* species, particular attention is often given to the UTL of eggs—the most thermal‐resistant life stage (Chakraborty et al. 2025; Dickerson 2007; Edillo et al. 2022; Kramer et al. 2020, 2021; Mulla and Chaudhury 1968). However, systematic testing across species and life stages under standardized heatwave conditions remains limited. Recent studies have examined heatwave responses across life stages in *Ae. albopictus* and in adults of *Ae. aegypti*, while comparable investigations are still lacking for *Cx. pipiens*. Importantly, none of these studies investigated how heatwaves may shape the potential distribution of these species (Alfaro et al. 2026; Covey et al. 2025; Dobbs et al. 2026; Hong et al. 2022; IPCC 2022). Addressing this gap is essential for improving mechanistic models that forecast vector distribution and disease risk under climate change scenarios. Survival outcomes under heatwave‐like exposures, analyzed across life stages, remain insufficiently quantified and likely yield more ecologically valid estimates of thermal resilience than existing data derived from constant‐temperature conditions (approach a) or brief heat shocks (approach b). This is particularly relevant because, in natural settings, high temperatures typically occur as short‐term extremes rather than persist throughout an entire life cycle. Consequently, mechanistic models relying solely on UTLs measured at constant or acute thermal exposure may fail to capture the full extent of mosquito thermal resilience of these species under heatwave conditions.

Despite the growing public health concern of mosquito‐borne disease outbreaks, survival responses of the vector species to heatwave exposures remain poorly characterised, particularly across different mosquito life stages. To better understand life‐stage‐specific thermal resilience and how rising temperatures may affect mosquito occurrence in Europe under both current and future heatwave scenarios, we evaluated the effects of simulated heatwaves on thermal resilience in three medically most relevant mosquito species ( *Cx. pipiens* , *Ae. albopictus* and *Ae. aegypti* ). Our focus on Europe is motivated by the above‐global average rate of climate warming (van Daalen et al. 2022) and the rapidly rising incidence of locally acquired dengue cases, mediated by *Ae. aegypti* in Madeira and *Ae. albopictus* in Italy, France and Spain, alongside an increase of West Nile virus cases, predominantly mediated by *Culex pipiens* (ECDC 2025b; Farooq et al. 2025).

**FIGURE 2:**
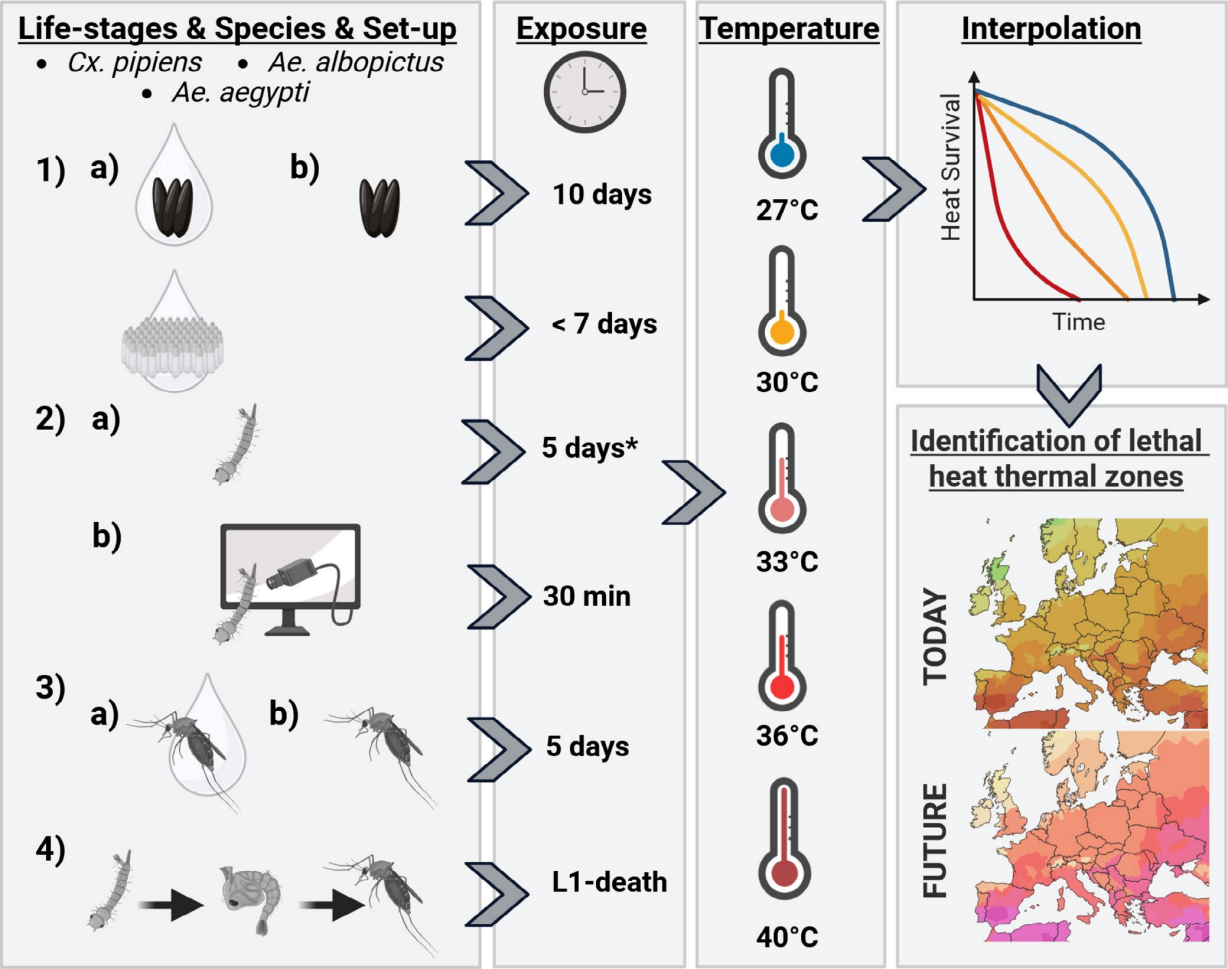
Summary of heat exposure experiments conducted with different lifestages of *Culex pipiens* , *Aedes albopictus* and *Ae. aegypti* providing input data for an experimental‐modelling framework. (1) Eggs of *Ae. aegypti* and *Ae. albopictus* were exposed to 27°C, 33°C, 36°C and 40°C under (a) high and (b) low humidity for 10 days, while *Cx. pipiens* eggs placed on top of water were exposed for up to 7 days. (2a) Larvae of *Cx. pipiens*, *Ae. albopictus* and *Ae. aegypti* were exposed to 27°C, 33°C, 36°C and 40°C for 5 days; specially, *Cx. pipiens* larvae were also tested at 30°C. (*) In addition, in a so‐called ‘recovery experiment’, *Cx. pipiens* larvae were exposed to 36°C for 24 h and 40°C for 1 h. (2b) Activity changes after heat exposure (27°C, 33°C, 36°C and 40°C) of *Cx. pipiens*, *Ae. albopictus* and *Ae. aegypti* larvae for 30 min were assessed. (3) Adults of *Cx. pipiens*, *Ae. albopictus* and *Ae. aegypti* were exposed to 27°C, 33°C, 36°C and 40°C for 5 days at (a) high and (b) low humidity; specially, *Cx. pipiens* adults were also exposed to 30°C but not to 40°C. (4) L1 larvae of *Cx. pipiens*, *Ae. albopictus* and *Ae. aegypti* were reared to adults until they died at 27°C, 30°C and 33°C, while *Ae. aegypti* was not tested at 30°C but 36°C. Egg, larval, and adult data were used for interpolation to identify lethal heat thermal zones in Europe under current and future climate change scenarios. Created in BioRender. Kramer, I. (2026) https://BioRender.com/6mcim3a.

In the present experimental‐modelling study, we aim to quantify life‐stage‐specific UTL in three arboviral vector species and project their potential persistence under present and future heatwave scenarios. To generate model input data (Figure 2), 5‐day heat events (27°C–40°C) were conducted on *Cx. pipiens*, *Ae. albopictus* and *Ae. aegypti* at the L3/L4 larval stage and as adults under low and high humidity. *Aedes* eggs were additionally exposed to 10‐day heat events and *Cx. pipiens* eggs for up to 7 days. Recovery potential was assessed by returning *Aedes* and *Cx. pipiens* eggs as well as *Cx. pipiens* larvae (after short‐term heat exposure at 36°C for 24 h and 40°C for 1 h) to standard conditions. For comparison with 5‐day heat events, full life‐cycle exposures at the same temperatures were conducted to align with existing literature. Survival was assessed over the full duration and after extreme exposure (after 5 days for larvae and adults, and from L1 to adult emergence in the life‐cycle experiment). Additionally, behavioural assays were used to detect heat‐induced sublethal effects. Life‐stage dependent UTLs were subsequently interpolated to extrapolate lethal thermal zones during heat events across Europe under current and future climate scenarios (SSP126, SSP370 and SSP585). The resulting ensemble mean maps based on annual and decadal averages across 10 CMIP6 models reveal spatiotemporal patterns of increasing thermal risk under different climate scenarios. These projections highlight regions where heat extremes are increasingly likely to exceed species‐specific UTLs, providing a basis to assess future persistence in three medical relevant mosquito species in Europe.

## 2 Material and Methods

### 2.1 Mosquito Material Used in the Experiments

Mosquito rearing for heat experiments was conducted in climate cabinets (CPS‐P530, RUMED, Germany) at the Merian Insectary, Institute of Tropical Medicine (ITM), Antwerp, Belgium. *Ae. aegypti* originated from Kathmandu, Nepal (collected Sept–Oct 2018; strain 20AAeg.NP‐GU.3; F12; MTA: 02.10.2019, Ref. No. 911); *Ae. albopictus* from Terni, Italy (July 2021; strain 21AAlb.IT‐GU.16; F5); and *Cx. pipiens* biotype *molestus* from Hove, Antwerp Province, Belgium (May–Sept 2020; strain 20CPip.BE‐ITMf.6; overlapping lab generations for 2.5 years).

*Cx. pipiens* larvae were reared in four trays (395 × 255 × 88 mm; 1.2–1.6 L water) with ~300 larvae each at 27°C, 80% RH, and a 16:8 h light: dark cycle. Hatching was induced in water, and larvae were fed ad libitum (L1/L2: TetraMin flakes; L3/L4: Koi sticks; Tetra, Germany). Pupae were transferred to 5 mL press‐on‐lid vials with ~2 mL water. Upon adult emergence, excess water was removed, leaving ~0.5 mL to maintain humidity (Table S1).

*Aedes* eggs were reared at 28°C, 80% RH, and a 16:8 h light: dark cycle. Larvae were kept in 1 L vessels (600 mL soft water; ~200 larvae each). Hatching was induced with a yeast–bouillon solution (0.150 g yeast and 0.150 g bouillon dissolved separately in 8 mL water; 1 mL of each added to 600 mL soft water). After hatching, larvae were maintained at 27°C, 80% RH, and a 16:8 h light: dark cycle until use in experiments.

### 2.2 Experimental Design of Heat Event Experiments

In total, seven heat event experiments (including a recovery experiment) were conducted with *Cx. pipiens, Ae. albopictus* and *Ae. aegypti* to assess thermal resilience across life stages (Figure 2). The effects of 10‐day heat events (27°C, 33°C, 36°C and 40°C) on *Aedes* eggs were tested under low (36.97%–48.89%) and high (75.09%–87.18%) humidity (details in Tables S1–S2). For *Cx. pipiens*, ≤ 7‐day heat events (27°C, 33°C, 36°C and 40°C) were analysed. Five‐day heat events (27°C, 30°C, 33°C, 36°C and 40°C) on larvae and adults under low (27%–42%) and high (82%–86%) humidity were compared among species (Table S1). Lifelong heat exposure (27°C, 30°C, 33°C and 36°C) was assessed by recording the duration from L1 stage to death. Short‐term (30 min) larval exposures (27°C, 33°C, 36°C and 40°C) were evaluated behaviourally, measuring distance moved (mm), acceleration (mm/s^2^), movement duration (s), and velocity (mm/s). Additionally, a recovery experiment with *Cx. pipiens* larvae exposed to 36°C (24 h) and 40°C (1 h) tested their ability to recover from heat stress.

Heatwave scenarios and test temperatures (27°C–40°C) were chosen based on published life‐cycle studies showing that *Cx. pipiens* did not survive at 33°C, *Ae. albopictus* tolerated up to 33°C (with limited data above 30°C), and *Ae. aegypti* survived up to 40°C (Kramer 2021; Moser et al. 2023). The maximum temperature tested for each species was set according to these species‐specific thermal limits. To avoid cross‐treatment interference, each temperature treatment was conducted in an individual incubator. Incubator temperatures (Reptile Egg Incubator, 25 L, VEVOR) were monitored with HOBO data loggers (MX1104, ONSET), and mean, minimum, and maximum values were calculated to verify actual heat exposure (Tables S2 and S3).

### 2.3 Heat Event Experiments With Three Mosquito Species

#### 2.3.1 Egg Response to Heat Events in an Either High or Low Humidity Conditions

Eggs of *Cx. pipiens* were exposed for up to 7 days to 27°C, 33°C, 36°C and 40°C. Eight to ten egg rafts per temperature were used, each egg raft less than 24 h old and individually placed in 100 mL cups containing 60 mL of soft water and a small amount of food (Tetra, Germany), as *Cx. pipiens* eggs are not desiccation‐resistant. If hatching occurred, larval survival was monitored for 24 h. After 7 days, unhatched egg rafts were transferred back to 27°C to allow for potential recovery, and survival was monitored for an additional 48 h.

Egg hatching success of the *Aedes* species was tested after 10 days of parallel exposure to 27°C, 33°C, 36°C and 40°C under high or low humidity. Eggs previously stored at 20.85°C and 80.73% RH were screened for integrity under a stereo microscope (25×; Stemi DV4, Carl Zeiss, Germany) before the experiment. For each temperature, two 1 L vessels were prepared, each containing four coffee filters (replicates) with 50 eggs each (*n* = 4). One vessel included a 50 mL water‐filled tube sealed with cotton to increase humidity (high humidity treatment; Table S2); the second vessel without the tube served as the low‐humidity treatment. Temperature and humidity were monitored using HOBO data loggers (MX1104, ONSET). Containers were wrapped in aluminium foil to maintain darkness. After 10 days, eggs from each replicate were transferred to individual 1 L vessels with hatching medium (as described for rearing), resulting in 32 vessels per species. Larvae were counted after 48 h and again after 96 h to determine hatching success. One outlier (*Ae. albopictus*, 36°C) was removed using the ROUT test (*Q* = 1%). Data normality was tested with the Shapiro–Wilk test. At 40°C, hatching was 0% in both species, preventing normality testing. As distributions varied across treatments, replicate means were compared per species using Kruskal–Wallis tests with FDR correction (Benjamini, Krieger, and Yekutieli; *Q* = 0.05). Thermal performance curves for *Ae. aegypti* under high and low humidity were analyzed using nonlinear regression (best‐fit model: ‘Beta growth then decay’; low humidity: *r* ^2^ = 0.45, Sy.x = 28.63; high humidity: *r* ^2^ = 0.70, Sy.x = 56.24). In *Ae. albopictus*, curve fitting was not sufficient. For *Cx. pipiens*, eggs either hatched before exposure ended or not at all; hence, no statistical analysis was performed. Statistical significance thresholds were *p* ≤ 0.05 (), *p* ≤ 0.01 (), *p* ≤ 0.001 () and *p* ≤ 0.0001 (****). All analyses were performed in Prism v11 (GraphPad Software Inc., USA).

#### 2.3.2 Larval Response to Heat Events

First, 8 day old L4 larvae of *Cx. pipiens*, *Ae. albopictus*, and *Ae. aegypti* were exposed in parallel to 27°C, 33°C, 36°C and 40°C for 5 days; *Cx. pipiens* was additionally tested at 30°C. For each temperature and species, eight larvae were randomly placed into five 100 mL cups (*n* = 5). Larvae were fed *ad libitum* with one Koi stick (Tetra, Germany) per day. Pupae were transferred to 5 mL press‐on‐lid vials containing ~2 mL water, and emerging adults were maintained with 0.25 mL water to ensure humidity. Survival and developmental stage were recorded after 5 h and daily up to Day 5; moving individuals were counted as alive. Survival over time was compared graphically and statistically among temperatures and species using Kaplan–Meier survival curves with log‐rank (Mantel–Cox) tests. When more than two groups were compared, multiple comparisons were corrected using the Holm–Šídák method, and *p*‐values were adjusted (*α* = 0.05). Larval development into pupae, female and male adults, or death under different heat treatments was also graphically compared.

To assess differences between populations not only over the entire experimental duration (Kaplan–Meier survival curves) of the heat events, but also to highlight larval survival after extreme heat exposure (i.e., maximum exposure duration tested), mean larval survival after 5 days was calculated for each heat event treatment. Percentage survival at Day 5 was first determined per replicate (up to 8 larvae per replicate), and the mean ± SD was then calculated across the five replicates (*n* = 5, details are provided in the Zenodo database). However, at 40°C, due to reduced lifespan, survival was separately assessed after 24 h instead. The normality of data gained from the heat event experiment with larvae (survival over time [24 h until 5 days] in %) was tested using the D'Agostino and Pearson test. As the majority of experimental data was not normally distributed Mann–Whitney tests were used to compare temperatures and species. *p*‐values (*α* = 0.05) were adjusted for multiple comparison using the Holm–Šídák method.

Second, in a recovery experiment for only the most heat‐sensitive species *Cx. pipiens*, larvae were exposed in parallel to 36°C for 24 h (*n* = 5; 8 larvae in each replicate, see details above) and 40°C for 1 h (*n* = 5; 8 larvae in each replicate, see details above) and immediately after exposure, placed back to 27°C. In this recovery experiment, survival, development time until reaching adulthood, and sex ratio were monitored.

Third, the activity of L3 and L4 larvae of *Cx. pipiens*, *Ae. albopictus* and *Ae. aegypti* was tested at 27°C, 33°C, 36°C and 40°C. Larvae were randomly distributed into four 4 mL‐well plates filled with water (*n* = 4 plates per species, 12 larvae each) and assigned to heat treatments for behavioural observation. Plates were exposed to each temperature for 35 min (5 min water acclimation, 30 min exposure), then returned to room temperature (~23°C) for 30 min. Activity patterns were recorded in darkness using the DanioVision chamber and EthoVision XT software (Noldus, Netherlands). For each larva, activity over 7 min was measured as distance moved (mm), velocity (mm/s), movement (s), and minimum/maximum acceleration (mm/s^2^). Data normality was tested using the D'Agostino and Pearson test. As most data were non‐normally distributed, differences between heat treatments within species and among species were analysed using nonparametric Mann–Whitney tests, with *p*‐values corrected for multiple comparisons via the Holm–Šídák method (alpha: 0.05).

#### 2.3.3 Adult Response to Heat Events at Low and High Humidity

The adults of *Cx. pipiens* were exposed in 5 mL glass vials to 27°C, 30°C, 33°C and 36°C under low (27%–42%) and high humidity (82%–86%) conditions. Individual adults of *Ae. aegypti* and *Ae. albopictus* were exposed in 5 mL glass vials to 27°C, 33°C, 36°C and 40°C for 5 days at low (27%–42%) and high humidity (82%–86%). To maintain high humidity in the high humidity treatment, adults in 5 mL glasses were kept with ~0.25 mL of water (Table S1). In contrast, the water was removed completely from the 5 mL glass vials in the low humidity treatment. At each heat event treatment (low and high humidity), up to 25 male and female adults (< 24 h old) were randomly tested (Table S4). Because adults did not emerge on the same day and mortality did not occur simultaneously, each individual mosquito was treated as an independent exposure run (=replicate). The survival of heat‐exposed adults was controlled after 5 h, 1, 2, 3, 4 and 5 days. Individuals that were still moving were counted as alive. The survival of the adults over time was analyzed as described for the larvae, but larvae were also compared with adults using the same method.

To assess differences under extreme heat exposure in adults, mean survival was calculated as for larvae (27°C, 30°C, 33°C and 36°C for 5 days; 40°C for 24 h). For each heat and humidity treatment, individual survival (*n* = 25) was recorded as 100% (alive) or 0% (dead) and mean ± SD was calculated for each duration (5 h, 1–5 days). Data normality was tested with the Shapiro–Wilk test on survival (%) over time. Differences between temperatures, humidity treatments, and species were analyzed using Mann–Whitney tests with Holm–Šídák correction (*α* = 0.05). Life‐stage differences (larvae vs. adults) were compared using the same approach.

#### 2.3.4 Heat Exposure During the Full Life‐Cycle of Mosquitoes

In the life‐cycle experiments, the L1 larvae of *Cx. pipiens* and *Ae. albopictus* were reared to adulthood until they died at test temperatures 27°C, 30°C and 33°C. The full life‐cycle thermal response of *Aedes aegypti* was tested at 27°C, 33°C and 36°C. In brief, the hatching of 200–300 eggs per species was induced simultaneously by placing them in yeast solution. After 48 h, five 100 mL cups, each with eight randomly selected larvae, were prepared for each temperature treatment (*n* = 5). Larvae were fed according to a feeding protocol (TetraMin flakes; Tetra, Germany; (Müller et al. 2013)). Pupae were individualized into 5 mL press‐on‐lid glasses filled with 2 mL water. Adults were kept in press‐on‐lid glasses containing 0.5 mL of water to maintain high humidity. Development was checked on a daily basis to record the sex of each individual, the development time, and the ELS per species in each different test temperature. The survival from L1 until adult emergence was checked daily. Individuals that were still moving were counted as alive.

To compare life‐cycle results with published data, mean survival from L1 to adult emergence and mean development time were calculated (literature: *Cx. pipiens* (Moser et al. 2023), *Aedes* (Kramer 2021)). Species were compared using nonlinear regression following Kramer et al. (2021), with outlier removal to account for differing experimental designs (survival model: lognormal; development model: second‐order polynomial [quadratic]; Table S5). Some datasets in Moser et al. (2023), were excluded due to unclear origins (Schrama et al. 2018; Zayed et al. 2019) or species uncertainty (Mpho et al. 2002). Development into life stages (pupa, female and male) and mortality under different temperatures were also graphically compared. Survival from L1 to pupation was compared across test temperatures and species using data from Kramer et al. (2021) and Vanslembrouck et al. (2024). Nonlinear regression (best‐fit model: centred second‐order polynomial [quadratic]; Table S5) was applied for interspecific comparison. Early life‐stage (ELS) data were analyzed using two‐way ANOVA with Tukey's post hoc test. ELS data at 27°C, 30°C and 33°C were normally distributed (D'Agostino and Pearson test) and analyzed accordingly, while data at 36°C were not and thus compared within *Ae. aegypti* using Mann–Whitney tests with Holm–Šídák correction (alpha = 0.05).

To assess differences under extreme heat exposure in the life‐cycle experiment, mean survival was calculated as the overall mean of replicates (L1 to adulthood). Survival data (%) were tested for normality using the Kolmogorov–Smirnov test. Temperature and species differences were analyzed with Mann–Whitney tests, and *p*‐values were corrected using the Holm–Šídák method (alpha: 0.05). Due to differing exposure durations between the life‐cycle and larval/adult experiments, means were not statistically compared.

### 2.4 Determination of Upper Lethal Thermal Limits

To identify areas of risk where the different mosquito species cannot survive heat events (27°C, 33°C, 36°C and 40°C), the UTL (=days of survival) at different temperatures was assessed across the experiments: (1) heat event experiment with eggs, (2) heat event experiment with larvae, (3) heat event experiment with adults (only the high humidity treatment was used to identify potential UTL of adults), and (4) the life‐cycle experiment. For eggs (i.e., larval hatching success from eggs), survival over time was taken at a single time point. Consequently, we report here the highest temperature at which at least 10% of eggs hatched, however, *Culex pipiens* eggs were excluded from this analysis, as they either hatched during exposure (27°C and 33°C) or did not survive simulated heatwaves (36°C and 40°C). In the larvae and adult heat event experiments, not all individuals died within the experimental period of 5‐days. Therefore, to interpolate how long individuals would survive beyond the experimental duration, non‐linear regression analysis was conducted. For the life‐cycle experiment, the mean ELS was used as the measure of UTL. Regression models were selected based on the goodness‐of‐fit (e.g., *R* ^2^, Sy.x, 95% confidence intervals; Table S9). In addition, biological context was considered during model selection, for instance, models predicting survival dropping below 0% far beyond observed mortality times were disregarded, even if their statistical fit appeared favourable. For the purpose of interpolation, UTL were derived from the regression models at the point where survival rates dropped below 10%. The 10% threshold was chosen as the minimum viable proportion required to withstand an ongoing heat event. This criterion is consistent with thresholds used in conservation contexts to define critical population decline (Pérez‐Pereira et al. 2022) and is further supported by field evidence demonstrating recovery of *Ae. aegypti* populations after suppression to approximately 15% of their original size following the release of genetically modified mosquitoes (Evans et al. 2019). Accordingly, the 10% threshold represents a level at which populations are expected to withstand an ongoing heat event and retain the capacity to recover, thereby providing a biologically grounded link between experimentally derived survival responses and ecological persistence in the field. Humidity was not incorporated into the spatial projections for two reasons. First, available climate datasets provide air humidity, which often does not represent the microclimatic humidity conditions experienced by mosquitoes near surfaces and breeding habitats. Second, our experimental design did not quantify responses along a continuous humidity gradient, making it difficult to translate the experimental ‘high’ and ‘low’ humidity treatments into quantitative relative humidity thresholds for modelling. To ensure consistency, the mean of these interpolated UTL was used as the final estimate, rather than the upper or lower confidence limits. These approaches provided consistent and biologically meaningful interpretations of UTL data across all experiments.

### 2.5 Mapping Lethal Thermal Zones Across Europe

To assess the spatial and temporal risk of survival‐limiting heat events for mosquito species, we applied experimentally derived UTL (see Section 2.4) to gridded climate data under both current and projected future conditions. UTLs for each species were determined using the following criteria: (1) temperatures where development still occurs, (2) the highest temperature tolerable for the species, and (3) the interpolated UTL (=days of survival) across all experiments (larvae, adult, and life cycle) corresponding to the least sensitive stage, i.e., the stage with the longest survival duration including eggs. A lethal heat event was defined as a sequence of consecutive days during which the daily mean temperature equalled or exceeded a given threshold for a duration equal to or greater than the species‐specific UTL (see Section 2.4; Table S10). We used daily mean temperature data from the E‐OBS gridded observational dataset, version 30.0e (Cornes et al. 2018), covering the period 2014–2024 at a spatial resolution of 0.1°. For each grid cell across Europe, we calculated the number of lethal events per year based on UTL and averaged the results over the 10‐year period. These calculations produced baseline risk maps that identify areas currently exposed to heat conditions that are likely to exceed mosquito UTLs and therefore representing zones of recurrent extreme thermal stress rather than deterministic maps of extinction. To assess how heat risk may evolve under climate change, we applied the same event‐based approach to daily mean temperature projections from 10 General Circulation Models (GCMs) from CMIP6, following the Inter‐Sectoral Impact Model Intercomparison Project (ISIMIP) protocols (Frieler et al. 2017). The models included ACCESS‐CM2, BCC‐CSM2‐MR, CNRM‐CM6‐1, CanESM5, EC‐Earth3, GFDL‐ESM4, INM‐CM5‐0, IPSL‐CM6A‐LR, MIROC6 and MRI‐ESM2‐0. Future climate data were obtained at 0.5° spatial resolution, bias corrected and downscaled according to ISIMIP standards. Analyses were performed for decadal periods from 2020–2030 to 2090–2100 under three common socio‐economic pathways (SSP1‐2.6, SSP3‐7.0 and SSP5‐8.5). For each GCM, scenario and time slice, the number of lethal heat events was counted annually per grid cell and averaged over the decade. An ensemble mean across the 10 GCMs was then calculated to produce decadal risk maps for each scenario. To allow spatial and temporal comparability, we performed a zonal analysis to determine the number of grid cells within the study area that experienced at least one lethal heat event per year. This allowed consistent tracking of both event frequency and spatial extent across scenarios and time periods. All spatial data processing and climate analyses were carried out using the R programming environment and the Terra package (Hijmans 2025; R Core Team 2025). For each decade, the number of grid cells experiencing at least one lethal heatwave event per year was calculated. Future cell counts were scaled by 25 to match current grid resolution, while present cells remained unscaled. The resulting values were normalized by the total number of current grid cells to derive the percentage of affected cells per year. Percent changes relative to the present period were then calculated for each future decade, and results were visualized using Prism (Version 10, GraphPad Software Inc., USA).

## 3 Results

### 3.1 Survival Above Life‐Cycle Derived UTLs Is Possible

**FIGURE 3:**
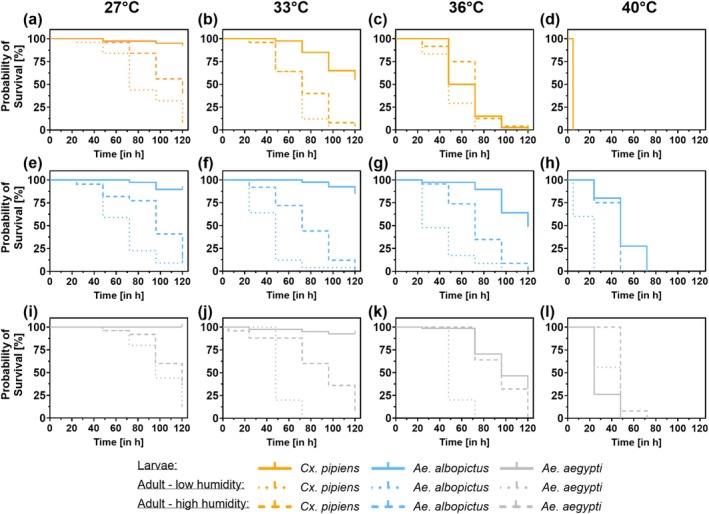
Mosquitoes' thermal resilience under simulated 5‐day heatwaves. Kaplan–Meier survival curves of *Cx. pipiens, Ae. albopictus*, and *Ae. aegypti* larvae and adults after exposure to 27°C (a, e, i), 33°C (b, f, j), 36°C (c, g, k) and 40°C for 5 days (d, h, l). Probability of adult survival has been assessed under low and high humidity conditions. Significant differences are given in Table S6. *Cx. pipiens* tested at 30°C is given in Figure S4.

Over the exposure duration of the heat event (duration: 0–5 days) and with increasing temperature (27°C–40°C), larvae of *Cx. pipiens* exhibited the lowest survival, followed by a larger gap by *Ae. albopictus* and *Ae. aegypti*. In the adult heat event experiment, between 33°C and 40°C, survival decreased from *Ae. aegypti > Ae. albopictus > Cx. pipiens*. At 33°C and 36°C, *Cx. pipiens* adults only survived heat events for less than 5 days (Figure 3, Figure S2, Table S6).

Neglecting *Aedes* eggs, larvae were the most resistant life stage to heat across all species and tend to survive longer than the adults, except at 40°C where *Ae. aegypti* adults exposed to high humidity survived slightly longer. Exposure of adults to high humidity compared to low humidity had a positive effect on survival across all species and heat events tested (Figures S3 and S4, Table S6). At low humidity, *Cx. pipiens* adults (33°C) and *Ae. albopictus* adults (33°C and 36°C) showed higher survival over heat event durations than *Ae. aegypti*. While at 33°C and 36°C at high humidity, survival is similar within all species (Figure 3, Table S6).

Within the tested larvae, the development to pupae or adults decreases with increasing heat events from 27°C to 40°C in all species. At 27°C and 33°C, development of larvae in all species occurs. Only *Ae. albopictus* and *Ae. aegypti* larvae developed at 36°C to further life‐stages, and no larvae developed to pupal stage at 40°C. In *Cx. pipiens*, larvae developed at 30°C, while at 33°C already many *Cx. pipiens* individuals died (Figures S4–S8).

**FIGURE 4:**
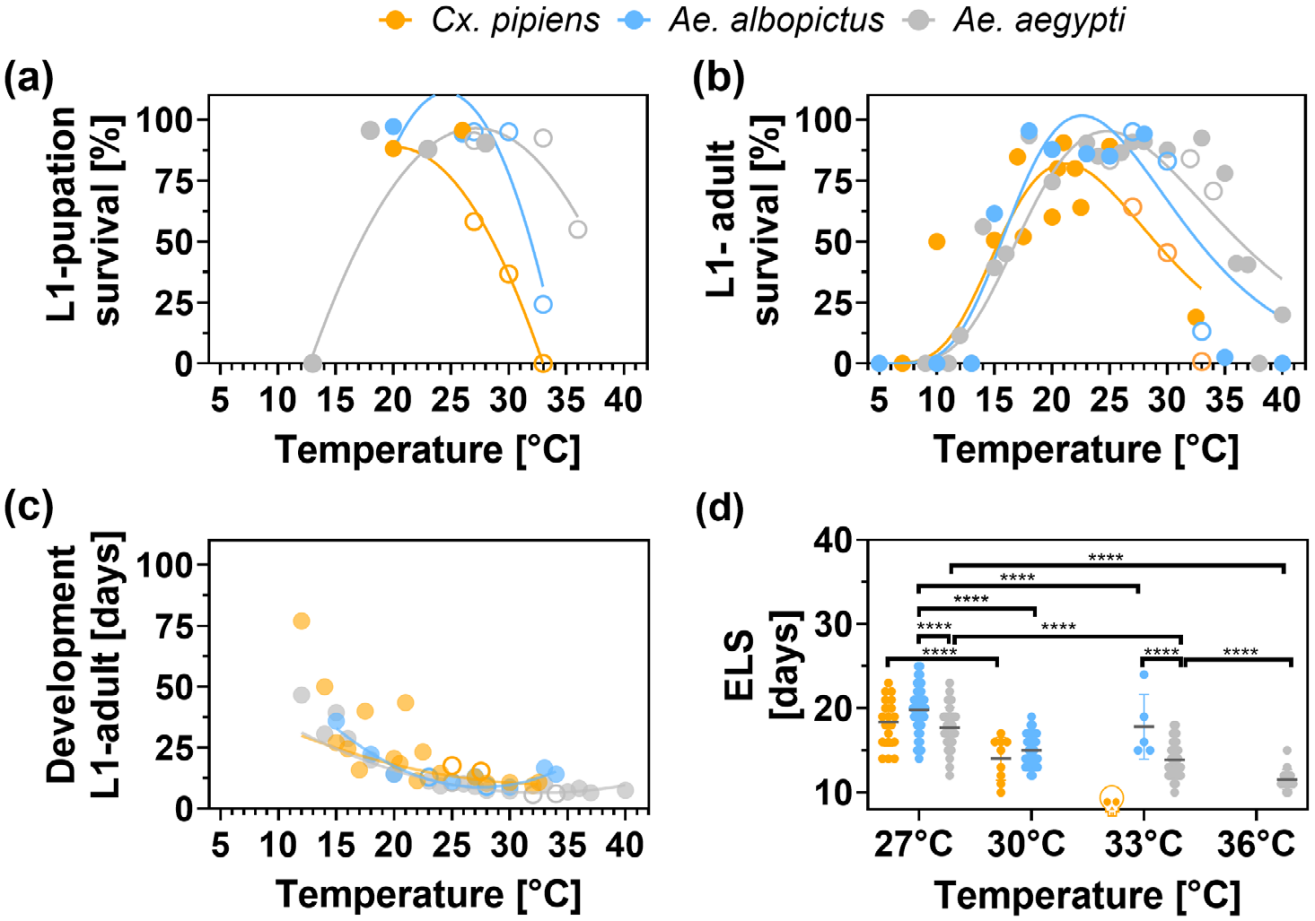
Thermal response of *Cx. pipiens*, *Ae. albopictus* and *Ae. aegypti* under constant temperature. Effect of temperature on survival, development and entire‐life span on *Cx. pipiens*, *Ae. albopictus* and *Ae. aegypti* is shown: (a) Survival from L1 to pupation in comparison with the same population of each species tested (Kramer et al. 2021; Vanslembrouck et al. 2024). (b) Survival from L1 to emergence of adults in comparison with other populations worldwide (Kramer 2021; Moser et al. 2023); (Table S5). (c) Development from L1 to emergence of adults in comparison with other populations worldwide (Kramer 2021; Moser et al. 2023); (Table S5). (d) Entire life span (ELS) from L1 until death from the life‐cycle experiment. Open circles in (a–c) indicate populations tested in the present life‐cycle experiment. More details including sex in Table S7.

In the life‐cycle experiment (duration: L1‐death), survival at high temperatures above 30°C decreases from *Ae. aegypti > Ae. albopictus > Cx. pipiens* (Figure 4A,B, Table S7). Here, development until adulthood occurs in all species at all tested temperatures, except at 33°C for *Cx. pipiens*. Increasing temperature reduced development to adulthood by more than 50% at 30°C in *Cx. pipiens* and at 33°C in *Ae. albopictus* (Figure S8). In a comparison with worldwide published literature, specifically for *Cx. pipiens* and *Ae. albopictus*, the conducted life‐cycle experiments fill a data gap (Figure 4B). With increasing temperatures, survival decreased in all three species (Table S7, Figure 4A,B), accompanied by a reduction in development time (Figure 4C) and a decline in the entire life span (ELS; Table S7; Figure 4D).

While in the life‐cycle experiment, survival above 33°C is low in *Cx. pipiens* and *Ae. albopictus* (Figure 3B), the larval heat event experiment shows that survival between 30°C and 36°C is still high for some days (Figure 3). Especially in *Cx. pipiens* and *Ae. albopictus*, the heat exposure over their entire life‐cycle (from L1 onwards) has negative effects on their development (Figures S7 and S8).

### 3.2 Extreme Heat Exposure Above 30°C Leads to Species‐ and Stage‐Specific Declines in Survival

At the point of extreme exposure (after 5 days of heat exposure), larval survival significantly decreases in all test species at heat events above 30°C (Figure S9A). Survival in *Cx. pipiens* larvae was observed only up to 33°C for exactly 5 days, while in *Ae. aegypti* and *Ae. albopictus*, it persisted up to 36°C. The survival of larvae significantly decreases at 40°C after only 24 h from *Ae. albopictus > Ae. aegypti > Cx. pipiens* (Figure S9E). In comparison to the larvae, adult survival after extreme heat exposure is lower at all temperatures, and especially at low humidity (Figure S9, Table S8). After extreme heat exposure, at low and high humidity survival only occurred at lower heat events (27°C, 30°C; Figure S9B,C). The maximum tested temperature (40°C for 24 h) highlights the high adult survival of *Ae. aegypti* compared to *Ae. albopictus*, with high humidity being beneficial for adult survival in both species, but especially in *Ae. albopictus* (Figure S9F,G and Table S8). Temperature acclimation over the full life‐cycle (L1‐to‐death) compared to the larval experiment (L3 and L4, L1‐L2 were not exposed; acclimation only 5 days) had a detrimental effect with lower survival at high temperatures (33°C) in *Cx. pipiens* and *Ae. albopictus*. In contrast, in *Ae. aegypti* acclimation over the full life‐cycle had a positive effect on survival (Figure S9A–D).

### 3.3 Sublethal Effects From Heat Exposure

Test temperature has only a minor effect on larval behaviour and was only significant in *Cx. pipiens*. The velocity and the cumulative movement duration of *Cx. pipiens* larvae are significantly lower at 40°C compared to 27°C (Figure S10B,C). Moreover, the minimum and maximum acceleration of *Cx. pipiens* larvae are significantly different between 27°C and 33°C and between 33°C and 36°C.

However, larval behaviour differed between species. *Ae. aegypti* was significantly the most active species by moving the longest distance over exposure time and being the fastest species (Figure S10A,B). *Culex pipiens* was the species that moved significantly less compared to the other species (Figure S10C). The minimum acceleration was similar in *Cx. pipiens* and *Ae. albopictus*, but lower in *Ae. aegypti* compared to *Cx. pipiens* (Figure S10D). The maximum acceleration of *Cx. pipiens* larvae was significantly higher than in *Ae. aegypti* larvae, while in *Ae. albopictus* larvae that was only the case at 27°C and 36°C (Figure S10E).

### 3.4 Potential for Recovery From Heat Exposure

Heat resistance eggs from *Aedes* species may serve as egg bank because they could restock the population after a heat event lasting not longer than 10 days at 36°C. In *Cx. pipiens* eggs, within 48 h at 27°C and 33°C, more than 90% hatch. In contrast, at 36°C and 40°C, no hatching occurs even after transferring the eggs back to 27°C for recovery after 7 days of exposure. This indicates that *Cx. pipiens* eggs cannot persist as an egg bank to restock the population following a heat event.

The eggs of *Ae. albopictus* and *Ae. aegypti* survived heat stress up to 36°C for 10 days, although *Ae. albopictus* eggs survived 36°C for 10 days only at high humidity. In *Ae. albopictus*, the highest egg survival was present at high humidity and at 27°C. In *Ae. aegypti*, the highest egg survival was measured at 36°C and high humidity exposure (Figure 2, Figure S1). Especially in *Ae. aegypti*, high humidity significantly increased the survival of the eggs, following a non‐linear relationship (Figure 2, Figure S1).

To further assess the potential for recovery from heat exposure in *Cx. pipiens*, we exposed L3/L4 larvae to different thermal stress scenarios and monitored their subsequent development. Accordingly, in the *Cx. pipiens* recovery experiment, after a 24 h exposure to 36°C, 5% of *Cx. pipiens* individuals still developed to adults, while after the 1 h exposure to 40°C 92.5% survived and developed (Figure S7).

### 3.5 Climate Warming Expands Heat‐Induced Lethal Zones for Mosquitoes in Europe

**FIGURE 5:**
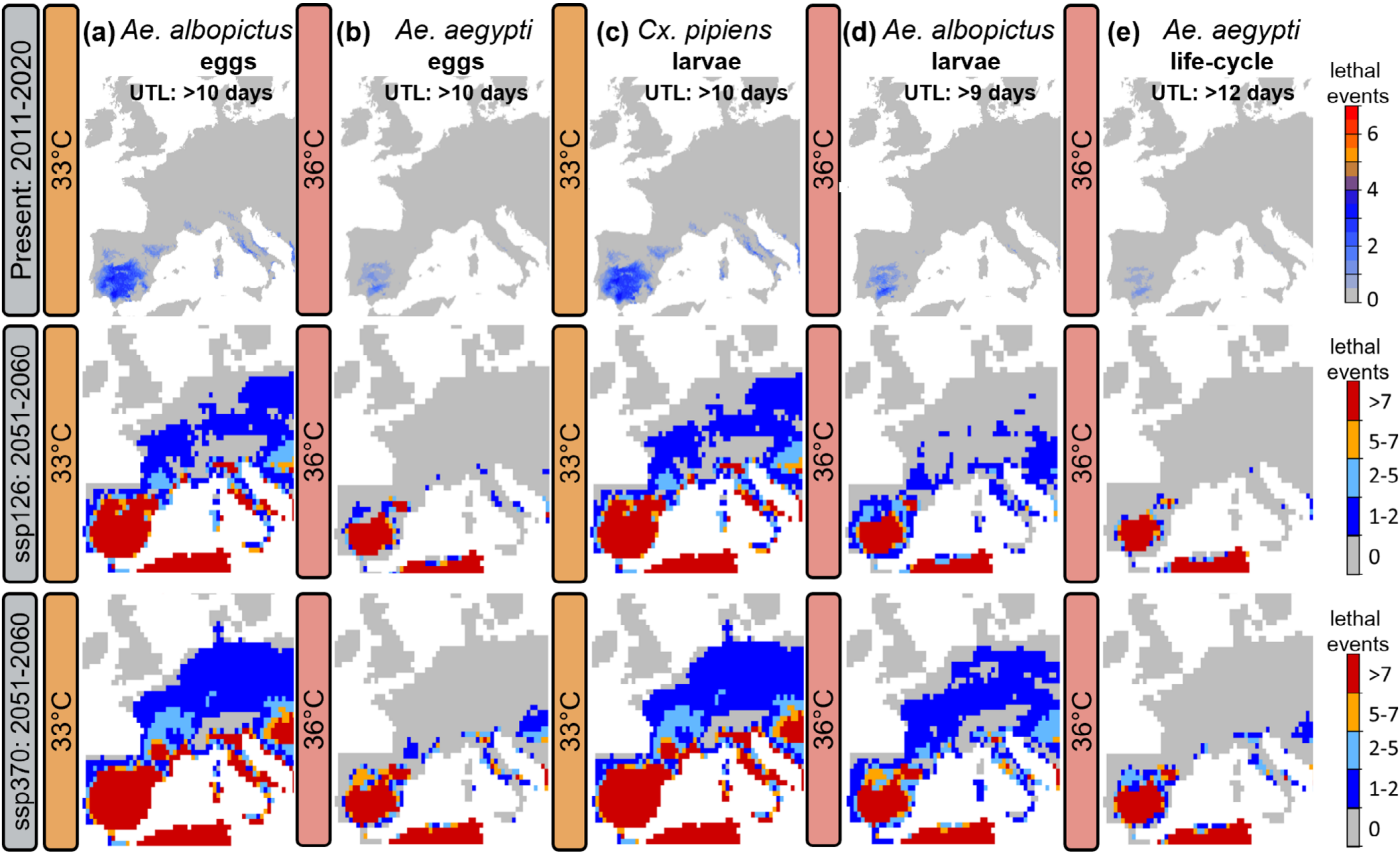
Projected lethal heat thermal zones in Europe for three mosquito species under current and future climate conditions (SSP126 and SSP370). Lethal thermal zones were projected across Europe for *Culex pipiens* , *Aedes albopictus* and *Aedes aegypti* under current and future climate conditions (SSP126 and SSP370; mean ensemble model is shown). Lethal events are defined as periods in which maximum daily temperatures exceed species‐specific upper thermal limits (UTL) in the most heat‐resistant life stages (Table 1), for a minimum number of consecutive days in the respective grid cell. Using this definition, we calculated the frequency and distribution of lethal events under current and future climate conditions to identify areas at risk of reduced survival. (a, b) UTL of *Aedes* eggs, (c) UTL of *Cx. pipiens* larvae, (d) UTL of *Ae. albopictus* larvae, (e) UTL of *Ae. aegypti* full life‐cycle. Interpolation details: Figures S11–S14, Table S9.

Survival interpolated for each life stage (larvae, adult) beyond the experimental duration, including the life‐cycle experiment, highlighted in all species prolonged survival, particularly in larvae and in individuals exposed over their full life‐cycle (life‐cycle experiment), compared to adults, with *Cx. pipiens* showing the lowest survival in general (Table S10, Figures S11–S14). Interpolated UTLs were used to identify heat‐induced lethal zones as areas of recurrent extreme thermal stress for the three mosquito species across Europe (Table 1, Figure 5).

**TABLE 1:** Species and life stage‐specific upper thermal limits (UTLs).

Lifestage	Species	Temperature	UTL [in days]
Eggs	*Aedes albopictus*	33	10
Eggs	*Aedes aegypti*	36	10
Larvae	*Culex pipiens*	33	10
Larvae	*Aedes albopictus*	36	9
Life‐cycle	*Aedes aegypti*	36	12

*Note:* UTLs were determined for each species using two criteria: (1) the highest temperature at which development still occurred, and (2) the highest experimentally tolerable temperature (survival ≥ 10%). Interpolated values are provided (see methods and Figures S11–S14). For eggs (=larvae hatching success form eggs), interpolation of survival over time was not possible due to measurements at only a single time point. Therefore, only the highest temperature at which at least 10% hatching occurred is reported. *Culex pipiens* eggs were excluded, as they either hatched during exposure (27°C and 33°C) or did not survive simulated heatwaves (36°C and 40°C).

In accordance, European regions currently experiencing extreme thermal stress for the three mosquito species *Cx. pipiens*, *Ae. albopictus* and *Ae. aegypti* are primarily expected to expand under all tested temperatures and climate change scenarios. Lethal thermal zones are projected to increase most notably for *Cx. pipiens*, followed *by Ae. albopictus* and *Ae. aegypti* (Figure 5).

**FIGURE 6:**
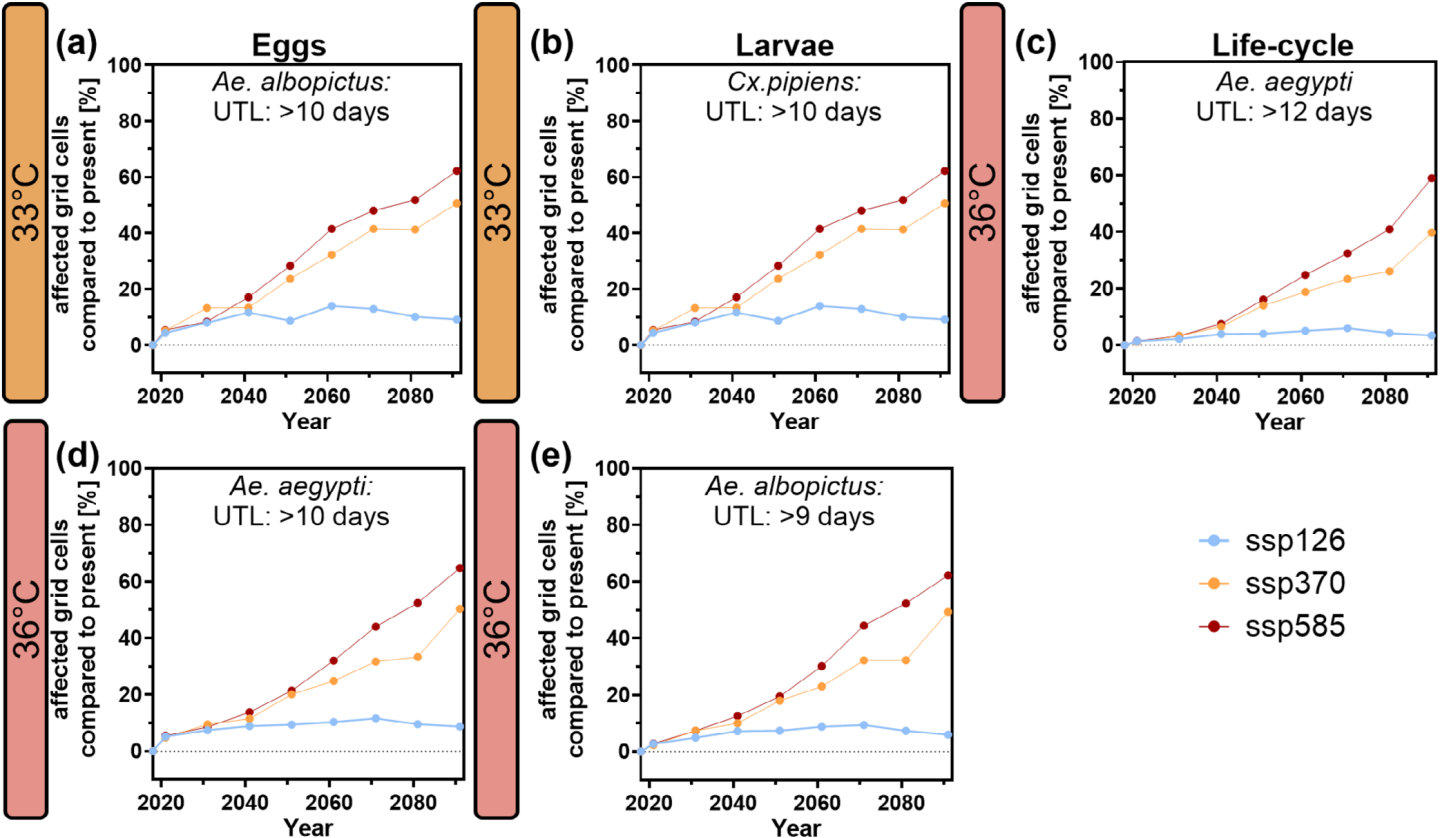
Projected increases in lethal heatwave events over time for three mosquito species in Europe. Shown is the percentage of grid cells experiencing at least one lethal heatwave event per year, relative to the present, under three SSP scenarios (SSP126, SSP370 and SSP585; ensemble model). Panels: (a, d) *Aedes* eggs; (b) *Cx. pipiens* larvae; (e) *Ae. albopictus* larvae; (c) *Ae. aegypti* full life cycle. Lethal events are defined as periods when maximum daily temperatures exceed the species‐specific upper thermal limits (UTL) of the most heat‐resistant life stage (Table 1). Changes are shown in 10‐year intervals from 2021 to 2100, with the present defined as 2014–2024 (dot at 2018). Interpolation methods: Figures S11–S14 and Table S9; detailed results for all life stages and temperature thresholds: Figures S15–S17.

Until 2100, under SSP370 and SSP585, risk areas are projected to increase across all species, heat events, and life stages (Figure 6, Figures S15–S17). In contrast, under SSP126, risk areas rise slightly at first but plateau and slightly decrease toward 2100, compared to the consistent increase seen in SSP370 and SSP585 (Figure 6, Figures S15–S17). However, the increase in high‐risk regions is more pronounced at higher temperatures (36°C and 40°C) compared to 33°C. While mosquito survival is shorter at higher temperatures, the expansion of extreme heat conditions under climate change scenarios from now until 2100 progresses more gradually at 33°C. This is because mosquitoes survive longer at 33°C, causing fewer time periods to surpass the critical threshold, while shorter survival at higher temperatures (36°C and 40°C) leads to more frequent threshold crossings and a faster expansion of regions experiencing extreme thermal stress (Figures 5 and 6).

## 4 Discussion

This study highlights how both life stages and combined environmental factors (humidity and temperature) are affecting the thermal resilience of three medically relevant mosquito species in Europe. Heat waves will majorly affect larvae and adults of all species, while both *Aedes* species will likely persist due to their egg bank, should *Ae. aegypti* become established. Our projections based on life‐stage specific upper thermal limits indicate that *Cx. pipiens*, due to its lower heat resilience, may experience a contraction in viable habitat under intensified heatwave conditions. In contrast, *Ae. albopictus* exhibits greater thermal resilience, suggesting a higher potential for persistence and for further geographic expansion in a warming Europe. For *Ae. aegypti*, projected range expansion is not limited by tested heatwave scenarios but rather by low humidity, reinforcing the hypothesis that aridity constrains its current distribution in Europe.

For *Cx. pipiens*, life‐cycle experiments reported that the species tends to tolerate temperatures only up to 33°C, *Ae. albopictus* up to 35°C, and *Ae. aegypti* up to 40°C (Kramer 2021; Moser et al. 2023). In the heatwave experiments performed herein, we demonstrated higher UTL than those reported in the literature. Accordingly, *Cx. pipiens* is able to withstand 36°C for more than 60 h in all of its life stages, while in *Ae. albopictus* and *Ae. aegypti*, survival was possible for up to 24 h at temperatures up to 40°C for all life stages (except for *Ae. aegypti* adults at low humidity levels). Yet, larval development in both *Ae. albopictus* and *Ae. aegypti* was only recorded at temperatures up to 36°C and in *Cx. pipiens* up to 33°C. In heat shock experiments, *Ae. aegypti* and *Ae. albopictus* can survive larval exposure to high temperatures (< 39°C) for up to 7 h, but at temperatures above 43°C, survival decreases rapidly and is shorter than 7 h (Sivan et al. 2019, 2021). The results describe here similarly highlight the larval heat resistance compared to the adults and the heat resistance of *Cx. pipiens* adults. In general, even short exposures of just 15 min to temperatures above 42°C negatively affect adult survival (Andersen et al. 2006). However, such acute heat shock experiment primarily define acute UTLs and may underestimate the chronic effect of heat stress, as demonstrated in the heatwave experiments described here. Previous studies on long‐term heat exposure of *Aedes* adult reported survival times of < 24 h at 38°C, whereas our study found survival of up to 3 days at 40°C. At 32°C, *Aedes* mosquitoes survived ~18 days, compared to ~5 days at 33°C in our experiments (Covey et al. 2025). These differences likely reflect both the strong impact of small temperature changes and variation in experimental design (e.g., rearing vessels vs. cage‐like enclosures), age of tested individuals (~48 h vs. < 24 h post adult emergence), and population background (field origin, number of laboratory generations). Our UTL estimates may appear conservative but likely approximate field conditions, as smaller containers may impose harsher environments similar to those in nature. Further research under ecologically realistic scenarios is needed to refine these UTL and further improve our understanding of thermal tolerance in these *Aedes* species.

Direct comparisons between UTLs derived from acute heat shock assays and those observed in our multi‐day heatwave experiments are inherently constrained by methodological and temporal differences in exposure regimes. Heat shock experiments, such as ramping tests with 1°C increments per minute or every 15 min from 26°C to 45°C, have indicated CT_max_ values of 43°C–44°C for adult *Ae. aegypti*, 44°C–45°C for *Ae. albopictus* larvae, 36°C–39°C for *Ae. albopictus* adults and below 43.5°C for *Cx. pipiens* adults (Gray 2013; Orlinick et al. 2023; Richardson et al. 2011). CT_max_ derived from life‐cycle experiments are in general lower compared to heat shock experiments (Couper, Nalukwago, et al. 2024). Our findings, based on repeated multi‐day sublethal heatwaves, yield intermediate CT_max_ values—higher than those from life‐cycle studies, yet lower than those from acute ramping tests. This discrepancy underscores the importance of aligning thermal tolerance metrics with ecologically relevant exposure scenarios when projecting species vulnerability under climate change.

Moreover, the potential for thermal acclimation also varied among species and depended on the type and duration of exposure. While full life‐cycle acclimation at high temperature had negative effects on *Ae. albopictus* and *Cx. pipiens*, it enhanced heatwave resilience in *Ae. aegypti* (Figure S9A,D). Positive acclimation effects have likewise been observed in heat shock experiments for all three species (Gray 2013; Ioannou et al. 2024; Sivan et al. 2017, 2019), although the mechanisms and ecological relevance of such plasticity may differ. Together, these findings underscore that both UTLs and acclimation responses are highly context dependent, and must be interpreted in light of species‐specific thermal history, experimental design, and relevant ecological timescales. Moreover, while we focused on how mosquitoes survive acute heatwaves, we must consider that mosquito species are able to adapt to rising temperatures over time and, potentially, exhibit higher heat resistance (in response to heatwaves) in the future. For instance, *Ae. aegypti* exhibited higher thermal resilience when reared over multiple generations at higher temperatures as also found in *Drosophila* (Chakravarti et al. 2017; Dennington et al. 2024). However, acclimation to high temperatures prior to heat exposure can also have negative effects on mosquitoes (Couper et al. 2025; Covey et al. 2025). In *Aedes sierrensis* , it was confirmed that the species can adapt to increasing temperatures faster than temperatures are increasing as a result of climate warming (Couper et al. 2025). This means that in addition to plastic responses to heat, mosquitoes may genetically adapt to withstand climate change‐induced high temperatures, allowing them to persist within their native habitats and not go extinct. While climate adaptation has been well studied in *Ae. sierrensis*, the potential for heat adaptation in *Ae. aegypti*, *Ae. albopictus* and *Cx. pipiens* requires further investigation, for example through population genomic studies on local thermal adaptation or multigenerational experiments on temperature response and acclimation. While all three of these species' climate adaptations have been investigated, the extent of these studies varies (Bennett et al. 2021; Carlassara et al. 2024; Haba et al. 2025; Kramer et al. 2022; Sherpa et al. 2019; Venkataraman et al. 2023).

Among *Aedes* species, eggs represent the most heat and desiccation‐resistant life stage. Our results confirmed that *Aedes* eggs remain viable for at least 10 days at 36°C. Eggs exposed to a constant temperature of 36°C at high humidity for 10 days still exhibited ~50% survival in our experiment. Despite the substantially shorter exposure duration relative to previous studies (Dickerson 2007; Edillo et al. 2022; Kramer et al. 2020, 2021; Mulla and Chaudhury 1968), survival remained within a comparable range. This pattern suggests that the apparent thermal tolerance of eggs may partly reflect their capacity to persist in a quiescent state, rather than active resistance to sustained heat stress. Under such conditions, embryonic development may slow or temporarily arrest while eggs remain viable, allowing them to withstand prolonged exposure to unfavourable temperatures. Consequently, survival measured after extended heat exposure may represent physiological persistence rather than true thermal tolerance of active developmental processes. This distinction is particularly relevant for *Aedes* eggs, which are known to exhibit substantial desiccation resistance and the capacity to remain viable for extended periods under environmentally stressful conditions. Such persistence mechanisms can buffer populations against short term environmental extremes by allowing eggs to survive until conditions again permit successful development and hatching. While fluctuating thermal regimes in the field may provide short recovery periods that could reduce cumulative heat stress, detrimental effects of temperature fluctuations become most pronounced when thermal variation approaches or crosses lower or upper developmental threshold temperatures. Thus, recent work indicates that heat exposure under fluctuating thermal regimes can still cause substantial damage to *Aedes* eggs, suggesting that their heat resistance may be lower than previously assumed (Alfaro et al. 2026; Eisen et al. 2014; Kramer et al. 2021). According to our results, the egg bank may buffer *Aedes* populations against the demographic impacts of recurrent heatwaves, contributing to their resilience in increasingly hostile climates. However, the capacity to withstand prolonged heat and desiccation merits further investigation if quiescent eggs function as a thermal refuge, enabling population persistence during extreme conditions. In contrast, *Cx. pipiens* eggs lack desiccation resistance, and while the adult stage is considered the most thermally tolerant life stage (Koenraadt et al. 2019), larvae in our heat experiments exhibited the highest thermal resilience combined with strong recovery potential. During heatwaves, *Cx. pipiens* adults will likely rely on behavioural thermoregulation such as seeking shaded areas or other cooler microhabitats as shelter to mitigate thermal stress. Similar behavioural avoidance of high temperature has been documented in adult *Ae. aegypti* (Verhulst et al. 2020). However, behavioural thermoregulation is not equally accessible to all life stages. While mobile adults may escape lethal surface temperatures, larvae lack such behavioural flexibility and are thus more vulnerable to extreme thermal events. *Aedes* mosquitoes—and to some extent *Cx. pipiens*—typically breed in small water containers (*Ae. aegypti*: < 5 L, *Ae. albopictus*: < 200 L, *Cx. pipiens*: < 2000 L but often < 200 L; Graziosi et al. 2020), in which water temperatures tend to be almost identical to ambient air temperatures. Even when eggs are laid in shaded environments, water temperatures can still vary and reach critical levels during heatwaves (Müller et al. 2018, Dalpadado et al. 2022). To assess larval stress responses under acute thermal stress, we exposed larvae to a brief (30‐min) heat event. Despite the brevity of the exposure, notable changes in behaviour were observed, particularly in the most heat‐sensitive species, *Cx. pipiens*, where larvae appeared to reduce activity possibly as a function of saving energy under heat stress (Figure S10). This differential life stage‐specific vulnerability of three mosquito species underlines the need to consider life stage‐specific UTLs and considering acclimation and thermoregulatory behavioural capacities when assessing species' resilience to increasingly frequent and intense heatwaves.

Current models of mosquito distribution rely on survival data from full life‐cycle experiments, not taking into consideration the effects of heatwaves or differential sensitivity of different life stages to varying temperatures (Couper, Nalukwago, et al. 2024; Mordecai et al. 2019). This study aims to fill this knowledge gap by providing life‐stage‐dependent survival data at heatwave conditions over a duration of up to 5 days especially for larvae and adults, offering a more natural estimate of thermal resilience. Our maps of heat‐induced lethal regions of the most heat resistance life‐stage align well with species current distribution patterns: *Ae. albopictus* is largely absent from areas in Spain identified as lethal zones, and the limited presence of *Cx. pipiens* in central Spain is consistent with our projections (EFSA 2013; Farooq et al. 2025; Mosquito Alert 2025; Withers et al. 2025). In contrast, *Ae. aegypti* is not yet established in Europe, with the exception of for Cyprus and Madeira, which were outside the scope of present study (ECDC 2025a). While climate‐based models project an expansion of climatically suitable regions for *Ae. albopictus*, *Cx. pipiens* and *Ae. aegypti* particularly in southern Europe (Pardo‐Araujo et al. 2024; Trájer 2021; Withers et al. 2025) our life‐stage specific models reveal a concurrent increase in heat‐induced lethal zones, in countries like Spain and Italy. Moreover, our approach, which incorporates experimentally derived, species‐specific UTLs, predicts lethal heat events for *Ae. aegypti* already under moderate scenarios (SSP370) by 2051–2060, whereas other models project declines in survival only under extreme scenarios (RCP8.5) by 2061–2080 (Trájer 2021) indicating that these models may overestimate the species' ability to establish and persist under climate change. Furthermore, our results indicate that *Ae. aegypti* is restricted by humidity rather than temperature alone, potentially explaining its absence in most parts of Europe due to low humidity (Drobinski et al. 2020). The role of humidity as a limiting factor certainly warrants greater consideration in future assessments of the species' potential distribution. Given its high heat resistance, an establishment of *Ae. aegypti* in the future in major parts of Europe may not be limited by heatwaves alone. In summary, distribution models typically neglect lethal thermal limits and life‐stage specific differences, relying primarily on climatic suitability or occurrence data. However, such models are not designed to detect UTL and may therefore overestimate future persistence (Athni et al. 2024).

Fluctuating temperatures were not tested in this study in order to ensure comparability with the majority of published literature (Kramer 2021; Moser et al. 2023). Although field conditions with temperature fluctuations can affect mosquito survival, development, and body size (Alam and Tuno 2020; Eisen et al. 2014; Richardson et al. 2011), other environmental stressors will likely counteract this effect. Constant‐temperature experiments, particularly when assessing thermal limits, provide realistic survival estimates that align with real‐world distribution limits (Athni et al. 2024; Shocket et al. 2025). Nonetheless, deviations in estimated heat tolerance at the *Aedes* egg stage highlights the need for complementary experiments under fluctuating conditions to better capture UTL, which are often hard to infer from distribution data alone (Athni et al. 2024). In the heatwave setup described here, life‐stage‐dependent phenotypic plasticity and thermal resilience were assessed in single populations of each species. However, geographical origins of field populations from other climates may influence survival to heat during heatwaves, as demonstrated or tested in other temperature response experiments (Carlassara et al. 2024; Dennington et al. 2024; Orlinick et al. 2023). To better understand the response of the mosquito to heatwaves and to quantify the plasticity of this trait within and between species, other populations from different climatic regions, along climatic clines as well as from different seasons should be tested since population and seasonal differences in plasticity to temperature‐response experiments are well known (Ioannou et al. 2024; Kramer et al. 2021; Oliveira et al. 2021).

Climate warming has been predicted to accelerate the spread of vector‐borne diseases, though its implications are complex (de Souza and Weaver 2024). While high temperatures can reduce transmission by lowering survival in mosquitoes and crossing arboviral thermal limits (Mordecai et al. 2019), they may also enhance transmission by accelerating viral replication and shortening the extrinsic incubation period (Mourya et al. 2004; Yadav et al. 2005; Zhao et al. 2010). Thus, the thermal plasticity of mosquito traits plays a critical role: UTLs differ between life stages as shown in the present study, UTLs have been reported to vary among populations (Carlassara et al. 2024; Dennington et al. 2024; Orlinick et al. 2023), and UTLs for infection often exceed those for survival, particularly at warm‐range edges (Couper, Nalukwago, et al. 2024; Mordecai et al. 2017). Behavioural responses to heat add additional complexity (Verhulst et al. 2020), and heat‐induced distributional shifts are likely. In summary, this study is the first to demonstrate how life‐stage‐specific responses to heatwaves shape lethal thermal zones that represent areas of recurrent extreme thermal stress and influence future mosquito survival and persistence, emphasizing that vector control strategies must adapt to these seasonal and spatial dynamics. *Culex pipiens* may decline in central Spain if selection pressures from extreme heatwaves intensify, although adults may persist by seeking shelter indoors during heatwaves. In contrast, *Aedes* species are expected to persist by shifting activity to more favourable seasons, supported by their resilient egg bank.

## Author Contributions

**Isabelle Marie Kramer:** conceptualization, formal analysis, writing – original draft, methodology, investigation, visualization, funding acquisition. **Stien Vereecken:** investigation, data curation, validation, writing – review and editing. **Adwine Vanslembrouck:** investigation, data curation, writing – review and editing. **Younes Smekens:** investigation, writing – review and editing, data curation. **Jacobus de Witte:** investigation, data curation. **Sofia Vielma:** investigation, data curation, writing – review and editing. **Aidin Niamir:** investigation, data curation, writing – review and editing, visualization, software. **Ruth Müller:** conceptualization, supervision, resources, project administration, writing – review and editing, funding acquisition.

## Funding

The publication was made possible with financial support from ‘ITM's SOFI programme supported by the Flemish Government, Science & Innovation’ and the DGD FA5 Synergy Fund (project ‘The impact of rapid CLIMate change on the Biodiversity‐health interface’ (CLIMB)) as well as from the National Research Platform for Zoonoses in Germany (HEAT project; Number 01Kl2312).

## Conflicts of Interest

The authors declare no conflicts of interest.

## Supporting information


**Figure S1:** Upper thermal limit of eggs. Mean egg survival (%, ±SD) of *Cx. pipiens*, *Ae. albopictus* and *Ae. aegypti* not normalized (A, B, C) and normalized (D, E) to 27°C (high humidity treatment) after an exposure to 27°C, 33°C, 36°C and 40°C. *Cx. pipiens* eggs were exposed for up to 7 days (eggs placed on water), while *Ae. albopictus* and *Ae. aegypti* eggs were exposed for 10 days at high or low humidity (not on water). *p* values for Kruskal–Wallis test are given. Non‐linear regression curves are given for *Ae. aegypti* (*p* < 0.0001).
**Figure S2:** Species comparison under simulated 5‐day heatwaves. Kaplan–Meier survival curves of *Cx. pipiens*, *Ae. albopictus* and Ae. aegpyti after an exposure to 27°C (A, E), 33°C (B, F), 36°C (C, G) and 40°C (D, H). (A–D) shows Kaplan–Meier survival curves for larvae. (E–H) shows Kaplan–Meier survival curves for adults (Table S6). Adult survival graphs were nudged to highlight differences between species.
**Figure S3:** Thermal resilience of *Cx. pipiens* under simulated 5‐day heatwaves. Kaplan–Meier survival curves of *Cx. pipiens* after an exposure to 27°C (A), 30°C (B), 33°C (C), 36°C (D) and 40°C (E). 30°C was added here because it is not present in Figure 3A.
**Figure S4:** Development of *Cx. pipiens* under simulated 5‐day heatwaves. Development of *Cx. pipiens* larvae as well as dead individuals after exposure to (A) 27°C, (B) 30°C, (C) 33°C, (D) 36°C and (E) 40°C for 5 days.
**Figure S5:** Development of *Ae. albopictus* under simulated 5‐day heatwaves. Development of *Ae. albopictus* larvae as well as dead individuals after exposure to (A) 27°C, (B) 33°C, (C) 36°C and (D) 40°C for 5 days.
**Figure S6:** Development of *Ae. aegypti* under simulated 5‐day heatwaves. Development of *Ae. aegypti* larvae as well as dead individuals after exposure to (A) 27°C, (B) 33°C, (C) 36°C and (D) 40°C for 5 days.
**Figure S7:** Life‐stages of *Cx. pipiens*, *Ae. albopictus* and *Ae. aegypti* larvae after 5 day exposure to 27°C, 30°C, 33°C, 36°C and 40°C. Development and number of dead individuals over the experimental duration are given in Figures S4–S6. In addition, *Cx. pipiens* development following exposure to 36°C for 24 h and 40°C for 1 h is presented as part of the recovery experiment.
**Figure S8:** Life‐stages of *Cx. pipiens*, *Ae. albopictus* and *Ae. aegypti* reached in the life‐cycle experiment after exposure to 27°C, 30°C, 33°C or 36°C.
**Figure S9:** Mean (±SD) survival (%) of *Cx. pipiens*, *Ae. albopictus* and *Ae. aegypti* after an extreme heat exposure to different temperatures (27°C, 30°C, 33°C and 36°C for 5 days and 40°C for 24 h). (A) Larval survival after an exposure to different temperatures for 5 days (*n* = 5 replicates with each 8 larvae). (B) Adult survival after an exposure to different temperatures for 5 days with low humidity (*n* = 20–25 adults). (C) Adult survival after an exposure to different temperatures for 5 days with high humidity (*n* = 20–25 adults). (D) Survival of individuals from L1 stage to adult (5 replicates with each 8 larvae; only including emerged adults). (E) Larval survival after an exposure to 40°C for 24 h (*n* = 5 replicates with each 8 larvae). (B) Adult survival after an exposure to 40°C for 24 h with low humidity (*n* = 20–25 adults). (C) Adult survival after an exposure to 40°C for 24 h with high humidity (*n* = 20–25 adults). Significant differences between larvae and adults (low and high humidity) are given in Table S8. Adjusted *p*‐values are given. Icons used were created with BioRender.com.
**Figure S10:** Analysis of activity parameters after heat exposure to 27°C, 30°C, 33°C, 36°C and 40°C for 30 min of *Cx. pipiens*, *Ae. albopictus* and *Ae. aegypti*. (A) Distance moved by the larvae [mm], (B) Velocity‐distance travelled by the larvae per unit of time [mm/s], (C) Cumulative duration of movement [s], (D) minimum acceleration [mm/s2], (E) maximum acceleration [mm/s2]. Adjusted *p* values are given.
**Figure S11:** Non‐linear regression models to interpolate upper thermal limits at 27°C. Non‐linear regression models were used to interpolate the time point at which 10% of mosquitoes (Cx. pipiens, *Ae. albopictus* and *Ae. aegypti*) still survived (upper thermal limit = ‘days of survival’) at 27°C, based on the tested larvae, adult (low and high humidity), and life‐cycle experiments. For fitting parameters see Table S9. Table 10 shows interpolated days at 10% survival threshold.
**Figure S12:** Non‐linear regression models to interpolate upper thermal limits at 33°C. Non‐linear regression models were used to interpolate the time point at which 10% of mosquitoes (Cx. pipiens, *Ae. albopictus* and *Ae. aegypti*) still survived (‘days of survival’) at 33°C, based on the tested larvae, adult (low and high humidity), and life‐cycle experiments. For fitting parameters see Table S9. Table S10 shows interpolated days at 10% survival threshold.
**Figure S13:** Non‐linear regression models to interpolate upper thermal limits at 36°C. Non‐linear regression models were used to interpolate the time point at which 10% of mosquitoes (Cx. pipiens, *Ae. albopictus*, and *Ae. aegypti*) still survived (‘days of survival’) at 36°C, based on the tested larvae, adult (low and high humidity), and life‐cycle experiments. For fitting parameters see Table S9. Table 10 shows interpolated days at 10% survival threshold.
**Figure S14:** Non‐linear regression models to interpolate upper thermal limits at 40°C. Non‐linear regression models were used to interpolate the time point at which 10% of mosquitoes (Cx. pipiens, *Ae. albopictus*, and *Ae. aegypti*) still survived (‘days of survival’) at 40°C, based on the tested larvae, adult (low and high humidity), and life‐cycle experiments. For fitting parameters see Table S9. Table S10 shows interpolated days at 10% survival threshold.
**Figure S15:** Projected increases in the proportion of affected grid cells over time for three mosquito species in Europe at 33°C. Shown is the percentage of grid cells experiencing at least one lethal heatwave event per year, relative to the present, under three SSP scenarios (SSP126, SSP370 and SSP585; ensemble model). Panels: Species‐specific upper thermal limits are for *Cx. pipiens*: larvae > 10 days (A), adults > 5 days (B), life‐cycle > 11 days (C); *Ae. albopictus*: larvae > 14 days (D), adults > 4 days (E), life‐cycle > 25 days (F); *Ae. aegypti* larvae: > 56 days (G), adults > 5 days (H), life‐cycle > 16 days (I). Changes are analyzed in 10‐year intervals from 2021 to 2100. Interpolation details: Figures S11–S14, Table S9.
**Figure S16:** Projected increases in the proportion of affected grid cells over time for three mosquito species in Europe at 36°C. Shown is the percentage of grid cells experiencing at least one lethal heatwave event per year, relative to the present, under three SSP scenarios (SSP126, SSP370 and SSP585; ensemble model). Panels: Species‐specific upper thermal limits are for *Cx. pipiens*: larvae > 3 days (A), adults > 3 days (B), life‐cycle > 0 days (C); *Ae. albopictus*: larvae > 9 days (D), adults > 4 days (E), life‐cycle > 1 days (F); *Ae. aegypti* larvae: > 7 days (G), adults > 5 days (H), life‐cycle > 12 days (I). Changes are analyzed in 10‐year intervals from 2021 to 2100. Interpolation details: Figures S11–S14, Table S9.
**Figure S17:** Projected increases in the proportion of affected grid cells over time for three mosquito species in Europe at 40°C. Shown is the percentage of grid cells experiencing at least one lethal heatwave event per year, relative to the present, under three SSP scenarios (SSP126, SSP370 and SSP585; ensemble model). Panels: (A) *Ae. albopictus* larvae (> 3 days), (B) *Ae. albopictus* adults (> 3 days), (C) *Ae. aegypti* larvae (> 2 days), (D) *Ae. aegypti* adults (> 3 days). Lethal events are defined as periods when maximum daily temperatures exceed the species‐specific upper thermal limits (UTL) given in Table 1. Changes are shown in 10‐year intervals from 2021 to 2100, with the present defined as 2014–2024 (dot at 2018). Interpolation methods: Figures S11–S14 and Table S9.

**Table S1:** Validation of low and high humidity in press‐on‐lid glasses. Test temperature = expected temperature also used in heat experiments. Temperature in the climate cabinet assessed with HOBO data loggers (type MX1104, ONSET). Humidity treatment = High humidity equals 1 mL of water present in the press‐on lid glass, while low humidity equals no water. Temperature = mean temperature measured with a temperature sensor (Thlevel) in press‐on lid glass over 1 h. Humidity = mean humidity measured with a humidity sensor (Thlevel) in press‐on lid glass over 1 h. Thevel loggers were controlled using with HOBO data loggers (type MX1104, ONSET).
**Table S2:** Mean, minimum and maximum temperatures (in °C) tested during heat experiments with eggs, larvae, adults and across the full life‐cycle, including rearing conditions. Temperatures within climate cabinets were tracked using HOBO data loggers (type MX1104, ONSET). Relative humidity in the adult experiment was set to low and high conditions, as described in Table S1, whereas during the life‐cycle experiment, adults were only exposed to high humidity (see Table S1).
**Table S3:** Mean, minimum and maximum temperature (in °C) and relative humidity (in %) tested during heat experiments with eggs. Relative Humidity and temperatures within climate cabinets were tracked using HOBO data loggers (type MX1104, ONSET). *Cx. pipiens* eggs were tested on water. Aedes eggs were exposed without water. The relative humidity gradually increased over the course of the measurement period, starting from the respective minimum temperatures.
**Table S4:** Amount of adults exposed to 27°C, 30°C, 33°C, 36°C and 40°C for 5 days.
**Table S5:** Fitting parameters of non‐linear regression models for temperature‐dependent survival and development time of mosquito species. Models were used to compare species. Details about raw data compared with the life‐cycle data originating from this study can be found in the literature review in (Kramer 2021). Due to experimental differences within the studies used for data extraction—such as variations in larval feeding—outliers were removed from the full dataset prior to performing non‐linear regression analysis (*Q* = 1%). Survival (%) and development (in days) from L1‐ adult emergence were analyzed worldwide for all populations were literature data was extracted from (Kramer 2021; Moser et al. 2023), as well as for the survival of the tested populations of this study (*Ae. aegypti*—Nepal, *Ae. albopictus*—Italy, *Cx. pipiens*—Belgium).
**Table S6:** Significance level of Kaplan–Meier survival curves for heat exposure experiments over the 5‐days lasting experimental exposure. (A) Larvae (L) of *Cx. pipiens*, *Ae. albopictus* and *Ae. aegypti* were exposed to 27°C, 33°C, 36°C and 40°C for 5 days, *Cx. pipiens* larvae were also tested at 30°C. (B) Adults of *Cx. pipiens*, *Ae. albopictus* and *Ae. aegypti* were exposed to 27°C, 33°C, 36°C and 40°C for 5 days at low humidity (A), *Cx. pipiens* larvae were also exposed to 30°C but not to 40°C. (C) Adults of *Cx. pipiens*, *Ae. albopictus* and *Ae. aegypti* were exposed to 27°C, 33°C, 36°C and 40°C for 5 days at high humidity (A–H), *Cx. pipiens* larvae were also exposed to 30°C but not to 40°C. (D) Adult: Comparison of the high and low humidity treatment and temperatures described in (B, C, E) Adult vs. larvae: Comparison of the adult compared to the larvae survival described in (A–C). (A–C) *p*‐values are given for Aedes in italic and for *Cx. pipiens* in regular. Adjusted *p*‐values are reported when more than two groups are compared (test: Holm–Šídák). ns = not signifiant, NA = not available because it was not tested. Icons used were created with BioRender.com.
**Table S7:** Life‐history parameters analysed after temperature exposure to 27°C, 30°C, 33°C or 36°C of *Cx. pipiens*, *Ae. albopictus* and *Ae. aegypti* from L1 until death. ELS = entire life span (days) in brackets number of individuals that survived until adult stage. L1‐adult = aquatic life span (days) in brackets individuals survived until emergence. Survival = survival of individuals until emergence compared to larvae still alive after 48 h. Sex ratio = male mosquitoes/sum of adult mosquitoes.
**Table S8:** Comparison of extreme heat exposure results (=maximum exposure duration tested, that is, mean survival after 5 days) of larvae vs. adult (high and low humidity). Groups were compared using Mann–Whitney tests including multiple comparison tests using the Holm–Šídák method to correct *p*‐values (alpha: 0.05). Adjusted *p*‐values are given.
**Table S9:** Fitting parameters of non‐linear regression models to interpolate upper thermal limits. Model and fitting parameters for best‐fitted curves using non‐linear regression to interpolate unknowns from standard curves within the heat exposure experiment, covering the full life cycle, larvae, and adults under low and high humidity conditions tested at 27°C, 33°C, 36°C and 40°C (see Table 1, Figures S11–S14).
**Table S10:** Species‐specific interpolated days until survival dropped below 10% at > 30°C. For the egg stage, only Aedes species with survival rates above 10% are shown, since Culex pipiens eggs either hatched during the exposure period or failed to survive. For adults low and high humidity are given but for maps and future predictions only the high humidity treatment was used. Details in Table S9 and Figures S11–S14. Whether development occurred was indicated based on Figures S7 and S8.

## Data Availability

The datasets generated during this study and the respective code is available in Zenodo: https://zenodo.org/records/17101066.
