# Supplementary material for: Heatwaves Constrain the Future Persistence of Mosquito Vectors in Europe
Source: Glob Chang Biol. 2026 Apr 27;32:e70876. doi: 10.1111/gcb.70876 (PMC13112340; doi:10.1111/gcb.70876)
Supplement: Supplementary file 1 — Figure S1: Upper thermal limit of eggs. Mean egg survival (%, ±SD) of Cx. pipiens, Ae. albopictus and Ae. aegypti not normalized (A, B, C) and normalized (D, E) to 27°C (high humidity treatment) after an exposure to 27°C, 33°C, 36°C and 40°C. Cx. pipiens eggs were exposed for up to 7 days (eggs placed on water), while Ae. albopictus and Ae. aegypti eggs were exposed for 10 days at high or low humidity (not on water). p values for Kruskal–Wallis test are given. Non‐linear regression curves are given for Ae. aegypti (p < 0.0001). Figure S2: Species comparison under simulated 5‐day heatwaves. Kaplan–Meier survival curves of Cx. pipiens, Ae. albopictus and Ae. aegpyti after an exposure to 27°C (A, E), 33°C (B, F), 36°C (C, G) and 40°C (D, H). (A–D) shows Kaplan–Meier survival curves for larvae. (E–H) shows Kaplan–Meier survival curves for adults (Table S6). Adult survival graphs were nudged to highlight differences between species. Figure S3: Thermal resilience of Cx. pipiens under simulated 5‐day heatwaves. Kaplan–Meier survival curves of Cx. pipiens after an exposure to 27°C (A), 30°C (B), 33°C (C), 36°C (D) and 40°C (E). 30°C was added here because it is not present in Figure 3A. Figure S4: Development of Cx. pipiens under simulated 5‐day heatwaves. Development of Cx. pipiens larvae as well as dead individuals after exposure to (A) 27°C, (B) 30°C, (C) 33°C, (D) 36°C and (E) 40°C for 5 days. Figure S5: Development of Ae. albopictus under simulated 5‐day heatwaves. Development of Ae. albopictus larvae as well as dead individuals after exposure to (A) 27°C, (B) 33°C, (C) 36°C and (D) 40°C for 5 days. Figure S6: Development of Ae. aegypti under simulated 5‐day heatwaves. Development of Ae. aegypti larvae as well as dead individuals after exposure to (A) 27°C, (B) 33°C, (C) 36°C and (D) 40°C for 5 days. Figure S7: Life‐stages of Cx. pipiens, Ae. albopictus and Ae. aegypti larvae after 5 day exposure to 27°C, 30°C, 33°C, 36°C and 40°C. Development and number of dead indivi [file GCB-32-e70876-s001.pdf]

Supplementary Figures

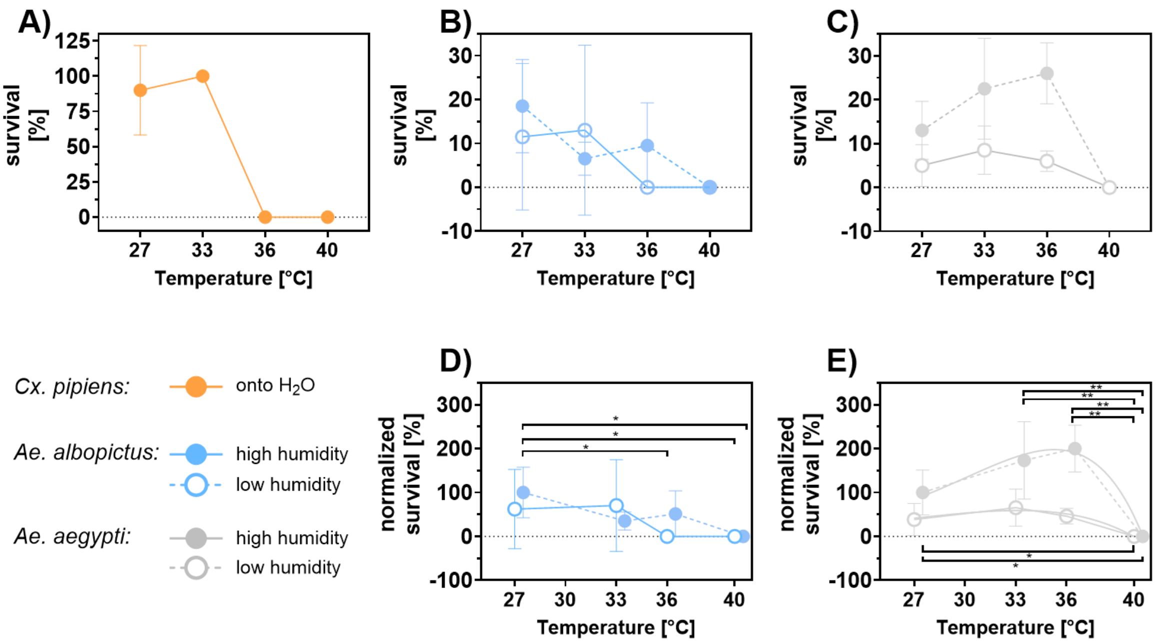

Supplement Figure 1. Upper thermal limit of eggs. Mean egg survival (%, ±SD) of *Cx. pipiens*, *Ae. albopictus* and *Ae. aegypti* not normalized (A,B,C) and normalized (D,E) to 27°C (high humidity treatment) after an exposure to 27°C, 33°C, 36°C and 40°C. *Cx. pipiens* eggs were exposed for up to 7 days (eggs placed on water), while *Ae. albppictus* and *Ae. aegypti* eggs were exposed for 10 days at high or low humidity (not on water). P values for Kruskal-Wallis test are given. Non-linear regression curves are given for *Ae. aegypti* (p <0.0001).

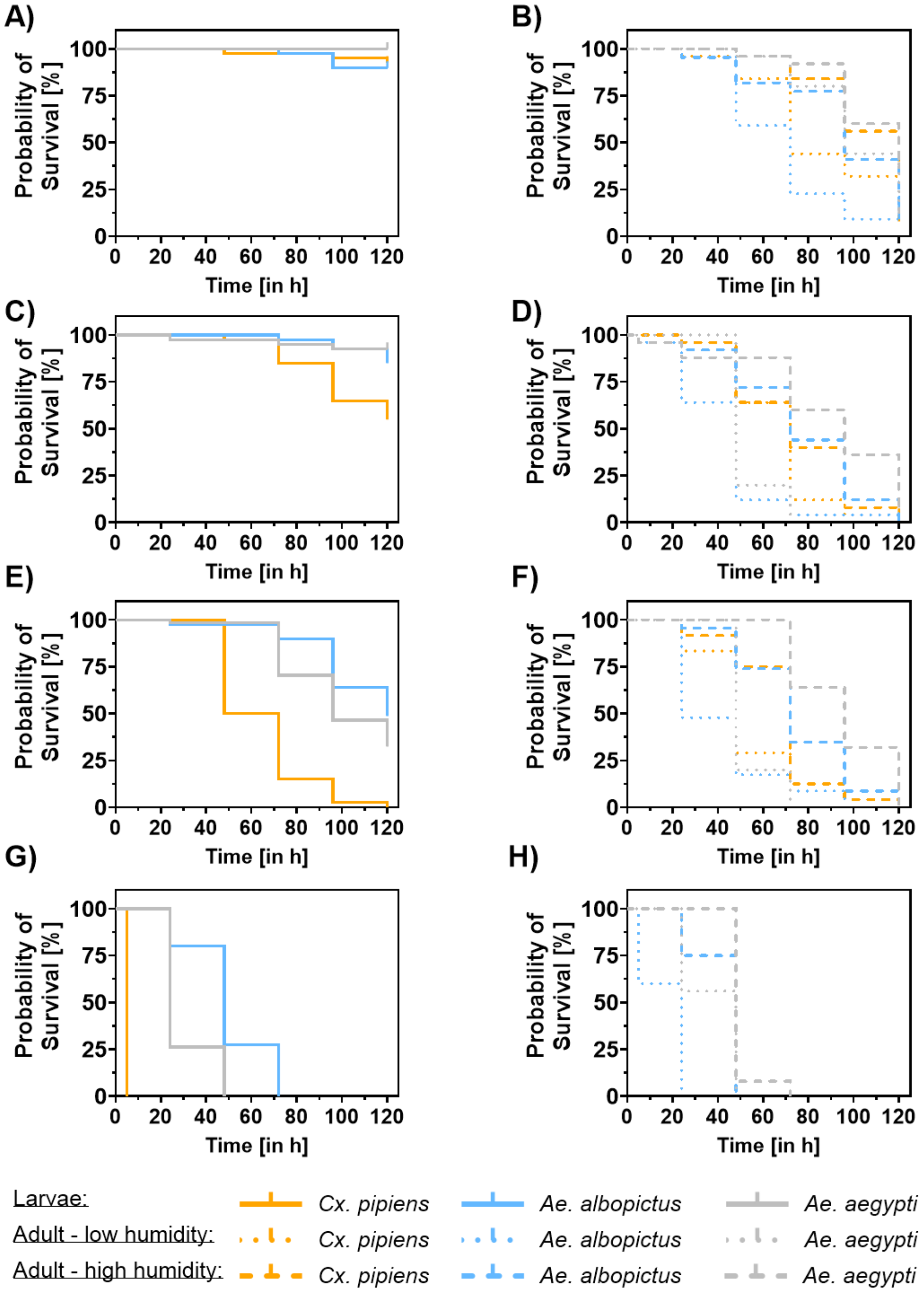

Supplement Figure 2. **Species comparison under simulated 5-day heatwaves.** Kaplan-Meier survival curves of *Cx. pipiens, Ae. albopictus* and *Ae. aegypti* after an exposure to 27°C (A, E), 33°C (B, F), 36°C (C, G) and 40°C (D, H). A-D) shows Kaplan-Meier survival curves for larvae. E-H) shows Kaplan-Meier survival curves for adults (Supplement Table 6). Adult survival graphs were nudged to highlight differences between species.

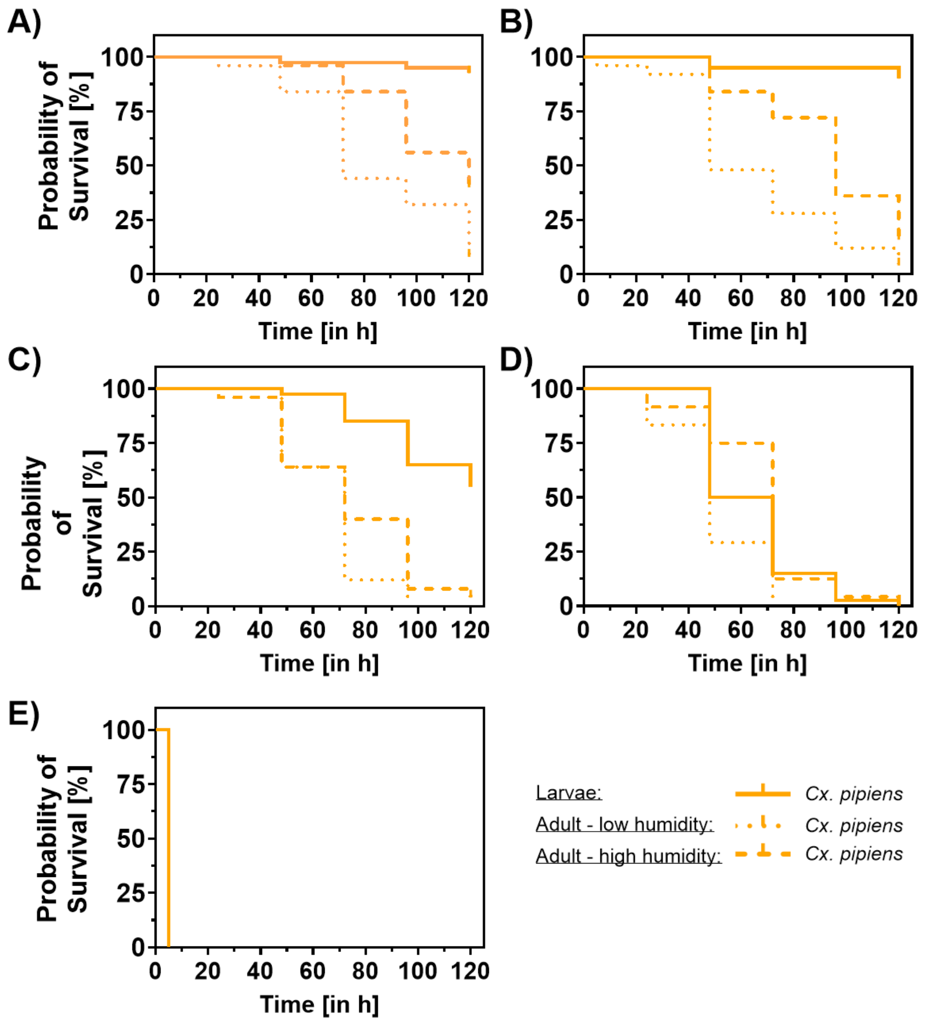

Supplement Figure 3. Thermal resilience of *Cx. pipiens* under simulated 5-day heatwaves. Kaplan-Meier survival curves of *Cx. pipiens* after an exposure to 27°C (A), 30°C (B), 33°C (C), 36°C (D) and 40°C (E). 30°C was added here because it is not present in Figure 3A.

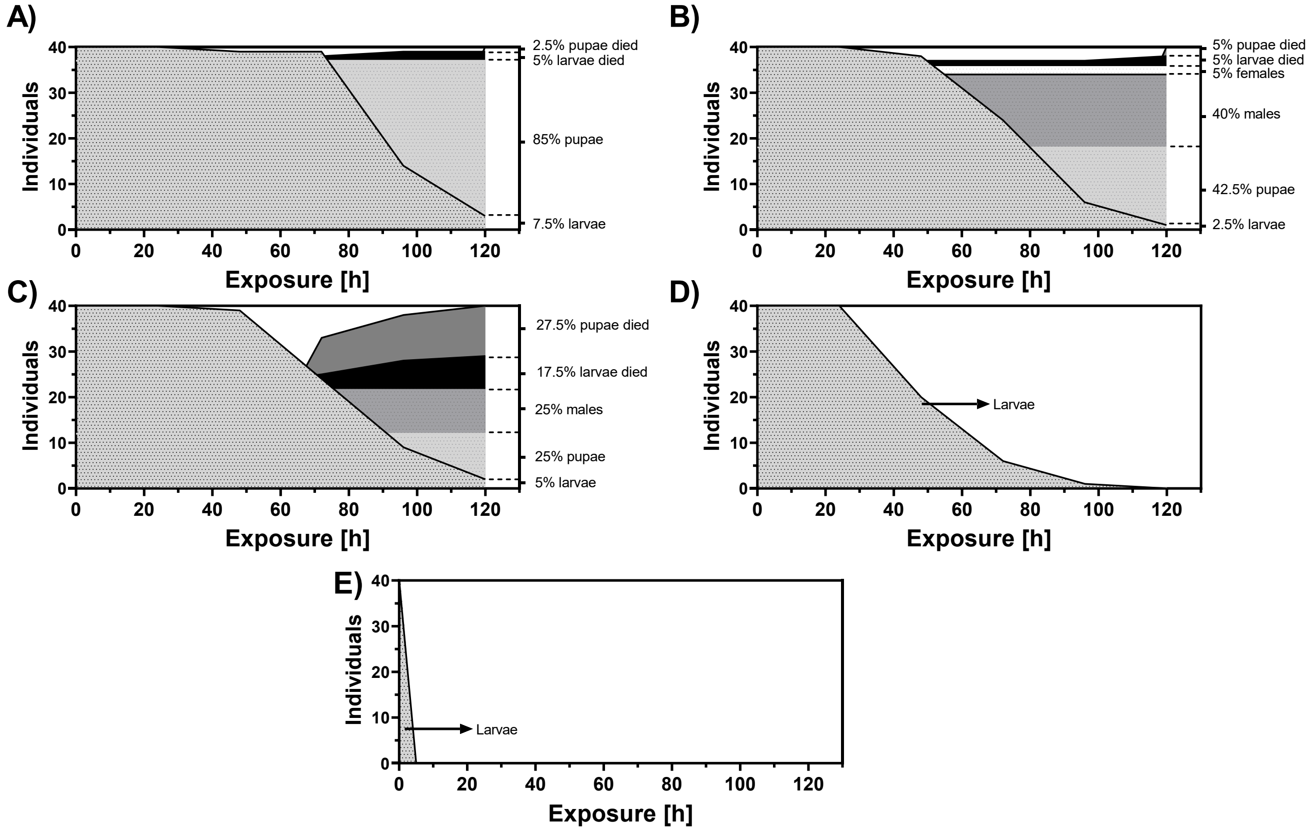

Supplement Figure 4. Development of *Cx. pipiens* under simulated 5-day heatwaves. Development of *Cx. pipiens* larvae as well as dead individuals after exposure to A) 27°C, B) 30°C, C) 33°C, D) 36°C, E) 40°C for 5d.

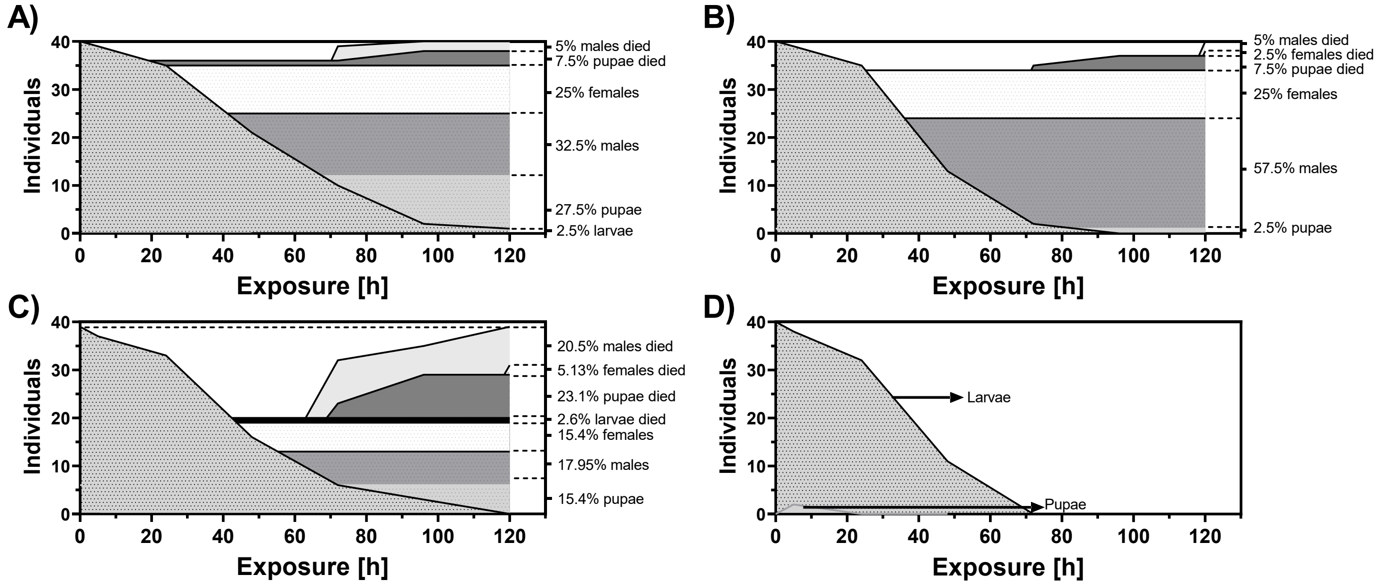

Supplement Figure 5. Development of *Ae. albopictus* under simulated 5-day heatwaves. Development of *Ae. albopictus* larvae as well as dead individuals after exposure to A) 27°C, B) 33°C, C) 36°C, D) 40°C for 5d.

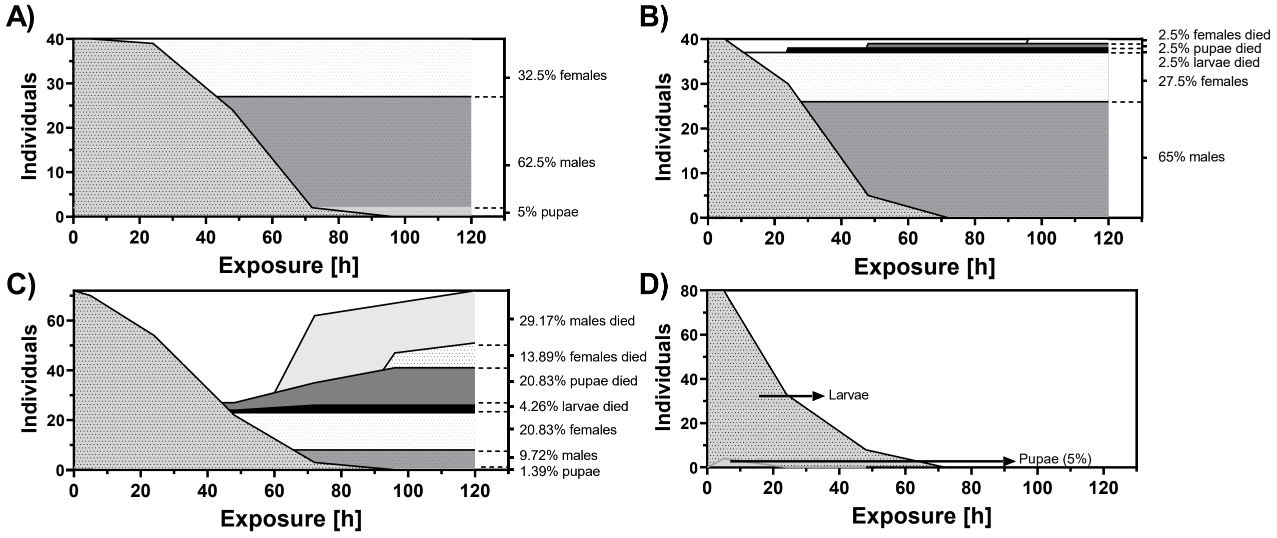

Supplement Figure 6. **Development of *Ae. aegypti* under simulated 5-day heatwaves.** Development of *Ae. aegypti* larvae as well as dead individuals after exposure to A) 27°C, B) 33°C, C) 36°C, D) 40°C for 5d.

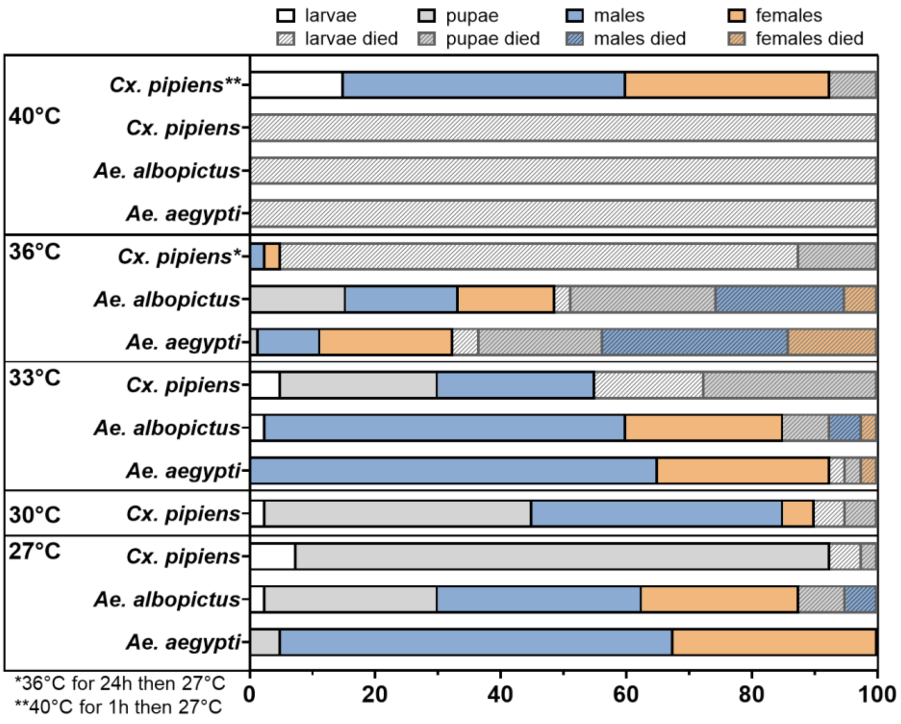

Supplement Figure 7. Life-stages of *Cx. pipiens, Ae. albopictus* and *Ae. aegypti* larvae after 5 day exposure to 27°C, 30°C, 33°C, 36°C, 40°C. Development and number of dead individuals over the experimental duration are given in Supplement figure 4, 5 and 6. In addition, *Cx. pipiens* development following exposure to 36 °C for 24 hours and 40 °C for 1 hour is presented as part of the recovery experiment.

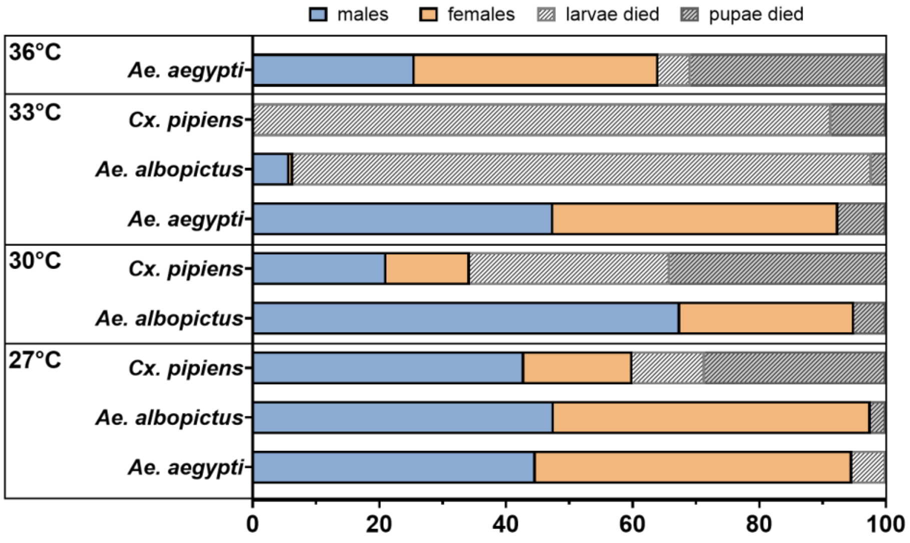

Supplement Figure 8. Life-stages of *Cx. pipiens, Ae. albopictus* and *Ae. aegypti* reached in the life-cycle experiment after exposure to 27°C, 30°C, 33°C or 36°C.

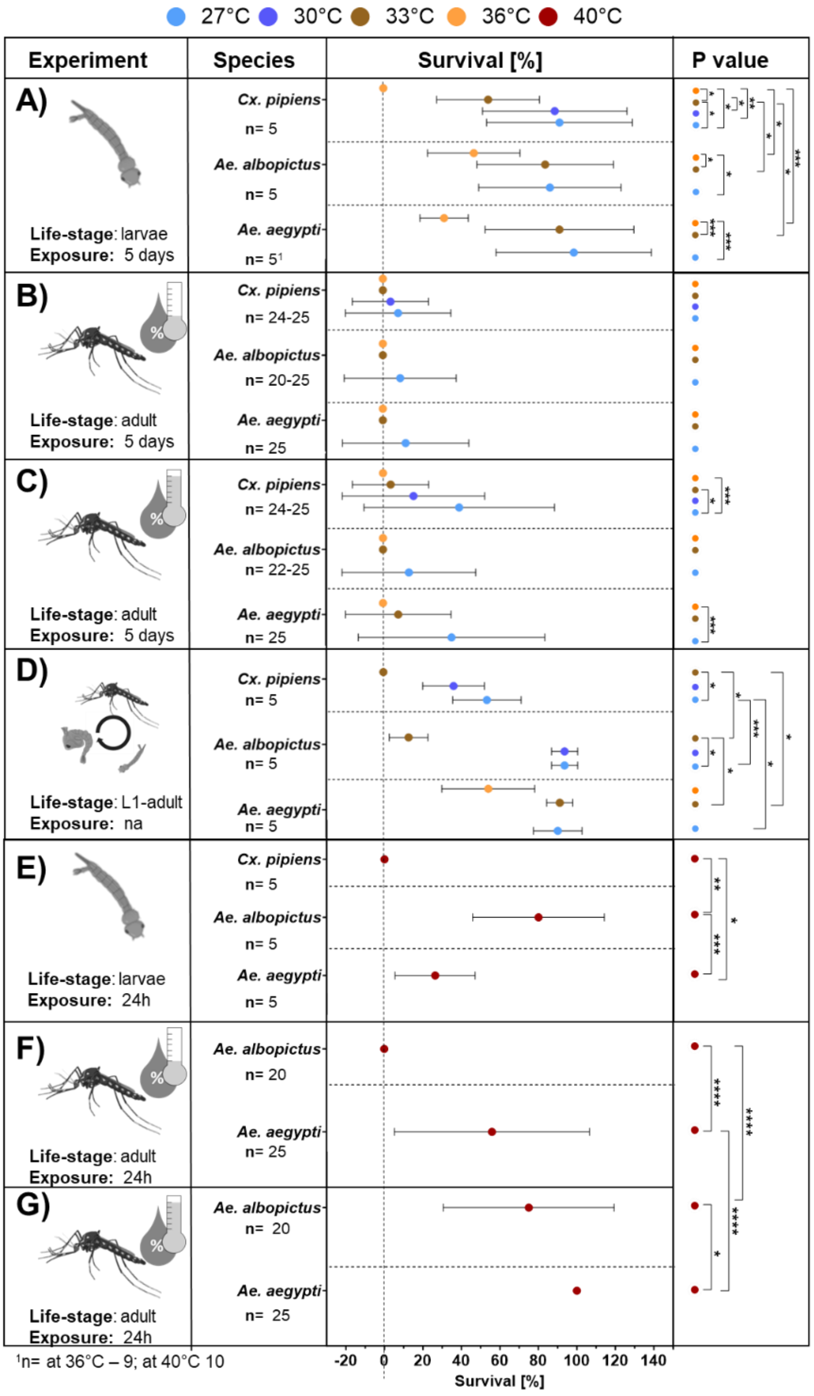

Supplement Figure 9. Mean (±SD) survival (%) of *Cx. pipiens*, *Ae. albopictus* and *Ae. aegypti* after an extreme heat exposure to different temperatures (27°C, 30°C, 33°C, 36°C for 5 days and 40°C for 24h). A) Larval survival after an exposure to different temperatures for 5 days (n = 5 replicates with each 8 larvae). B) Adult survival after an exposure to different temperatures for 5d with low humidity (n = 20-25 adults). C) Adult survival after an exposure to different temperatures for 5d with high humidity (n = 20-25 adults). D) Survival of individuals from L1 stage to adult (5 replicates with each 8 larvae; only including emerged adults). E) Larval survival after an exposure to 40°C for 24h (n = 5 replicates with each 8 larvae). B) Adult survival after an exposure to 40°C for 24h with low humidity (n = 20-25 adults). C) Adult survival after an exposure to 40°C for 24h with high humidity (n = 20-25 adults). Significant differences between larvae and adults (low and high humidity) are given in Supplement Table 8. Adjusted p-values are given. Icons used were created with BioRender.com.

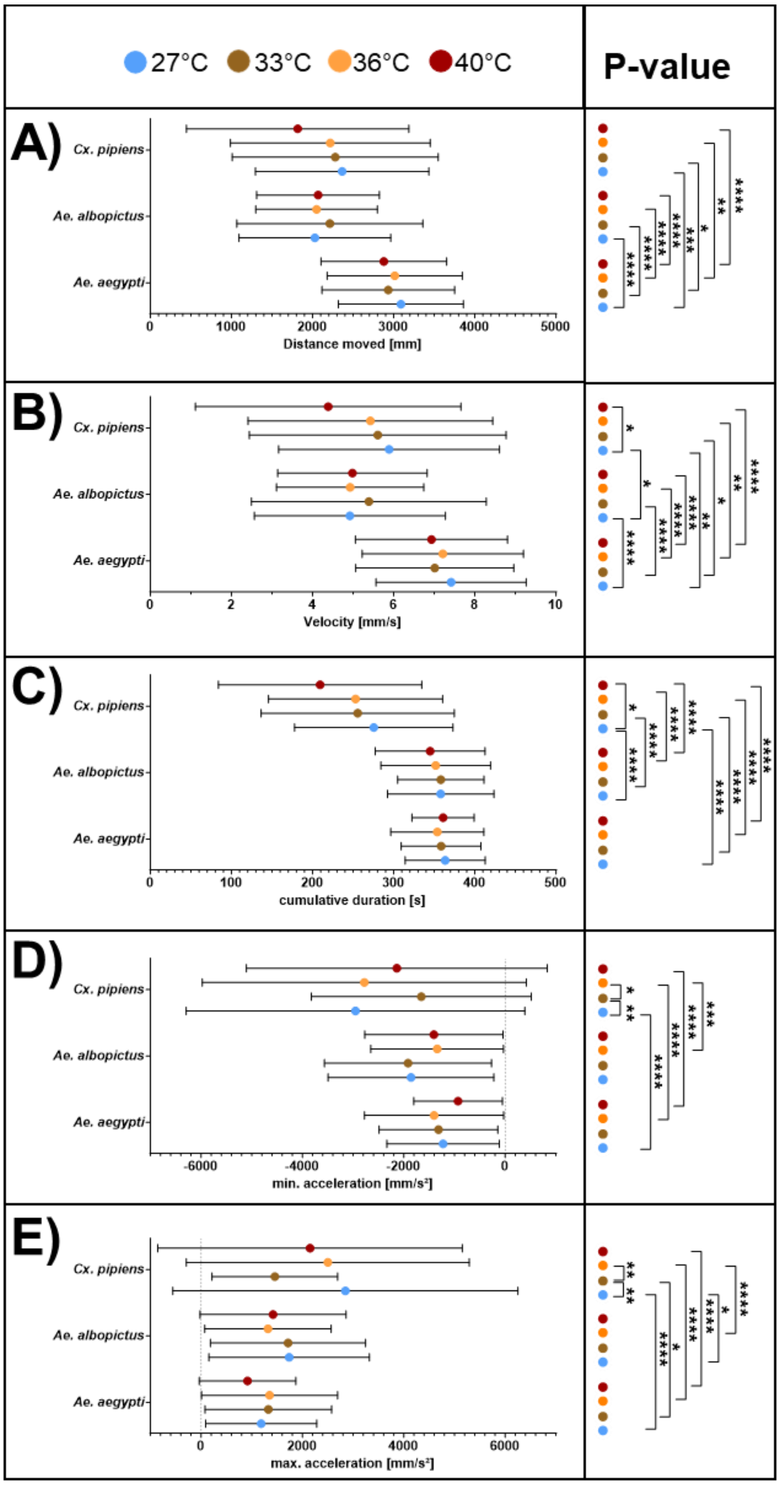

Supplement Figure 10. Analysis of activity parameters after heat exposure to 27°C, 30°C, 33°C, 36°C, 40°C for 30 min of *Cx. pipiens, Ae. albopictus* and *Ae. aegypti*. A) Distance moved by the larvae [mm], B) Velocity- distance travelled by the larvae per unit of time [mm/s], C) Cumulative duration of movement [s], D) minimum acceleration [mm/s^2], E) maximum acceleration [mm/s^2]. Adjusted p values are given.

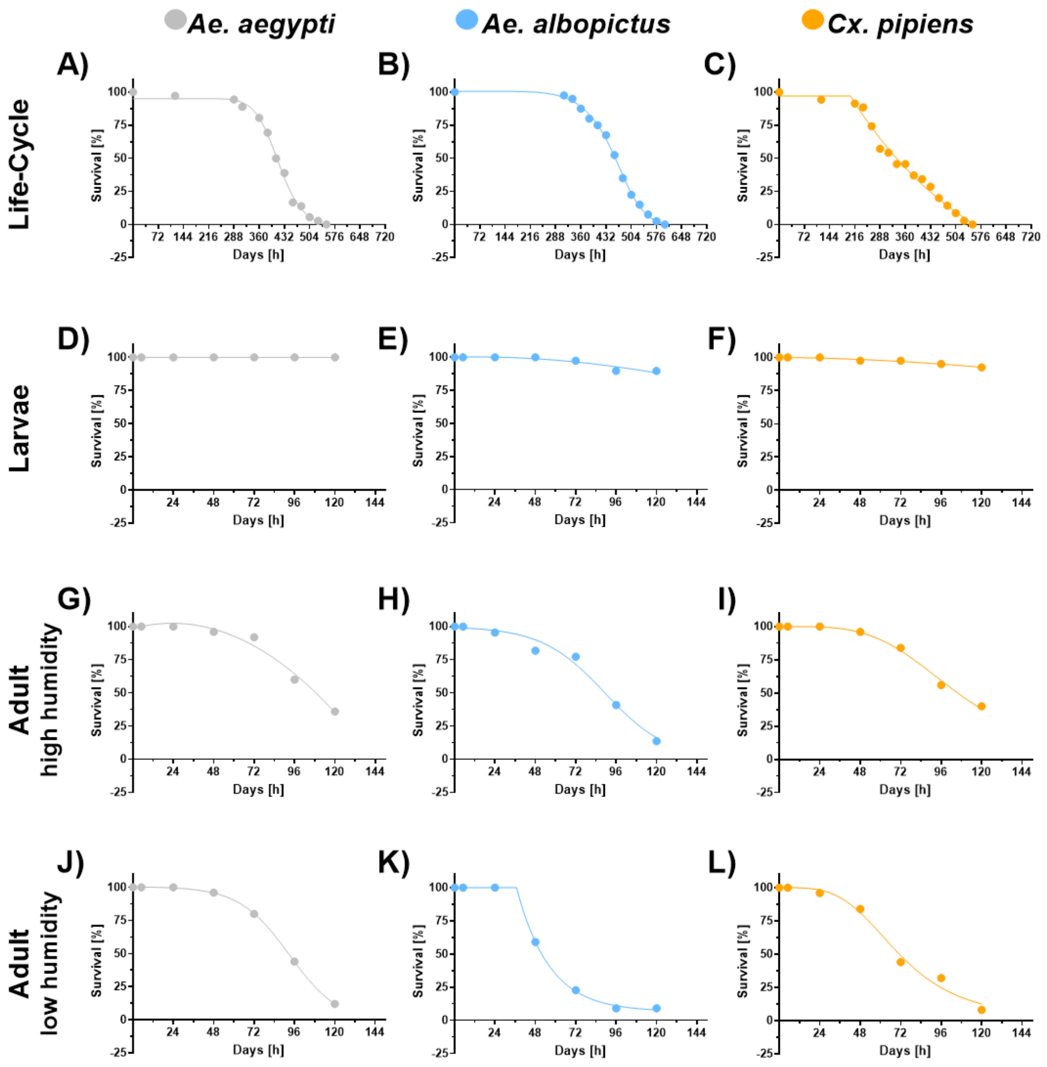

Supplement Figure 11. Non-linear regression models to interpolate upper thermal limits at 27°C. Non-linear regression models were used to interpolate the time point at which 10% of mosquitoes (*Cx. pipiens, Ae. albopictus*, and *Ae. aegypti*) still survived (upper thermal limit='days of survival') at 27°C, based on the tested larvae, adult (low and high humidity), and life-cycle experiments. For fitting parameters see Supplement Table 9. Supplement Table 10 shows interpolated days at 10% survival threshold.

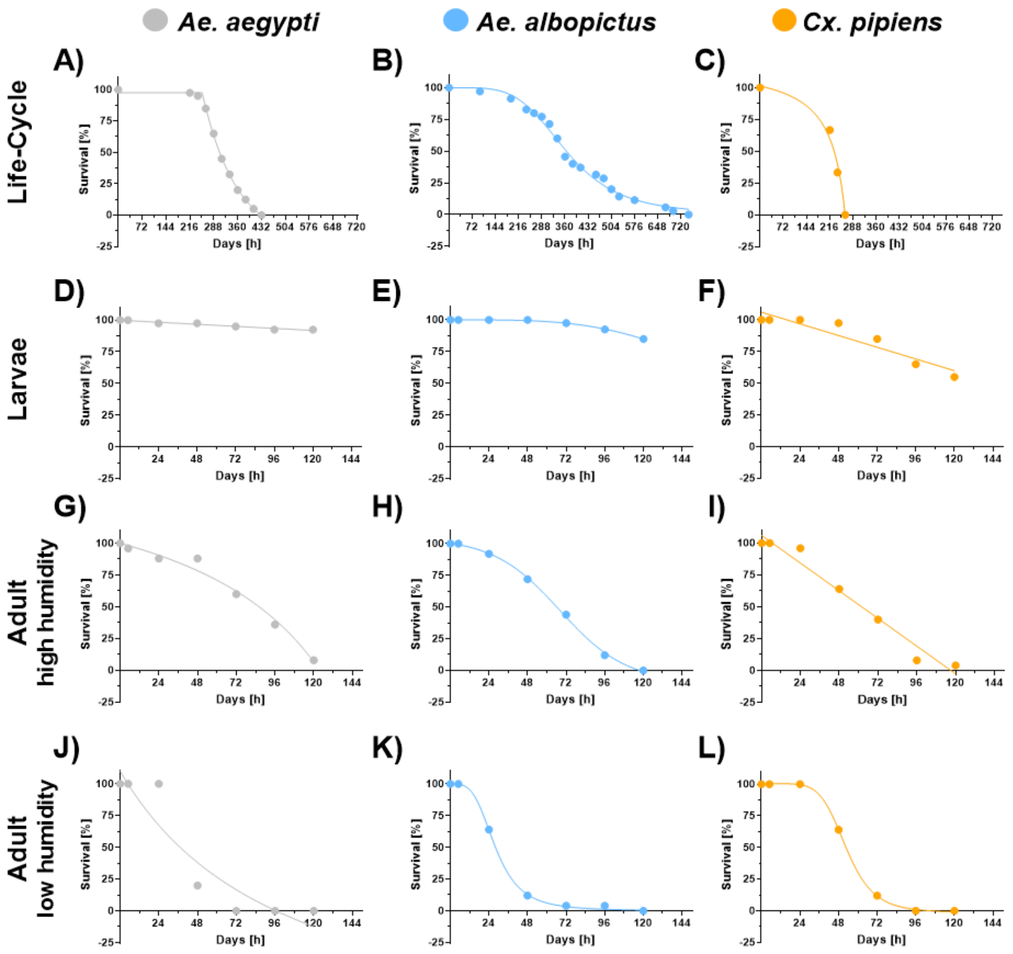

Supplement Figure 12. Non-linear regression models to interpolate upper thermal limits at 33°C. Non-linear regression models were used to interpolate the time point at which 10% of mosquitoes (*Cx. pipiens, Ae. albopictus*, and *Ae. aegypti*) still survived ('days of survival') at 33°C, based on the tested larvae, adult (low and high humidity), and life-cycle experiments. For fitting parameters see Supplement Table 9. Supplement Table 10 shows interpolated days at 10% survival threshold.

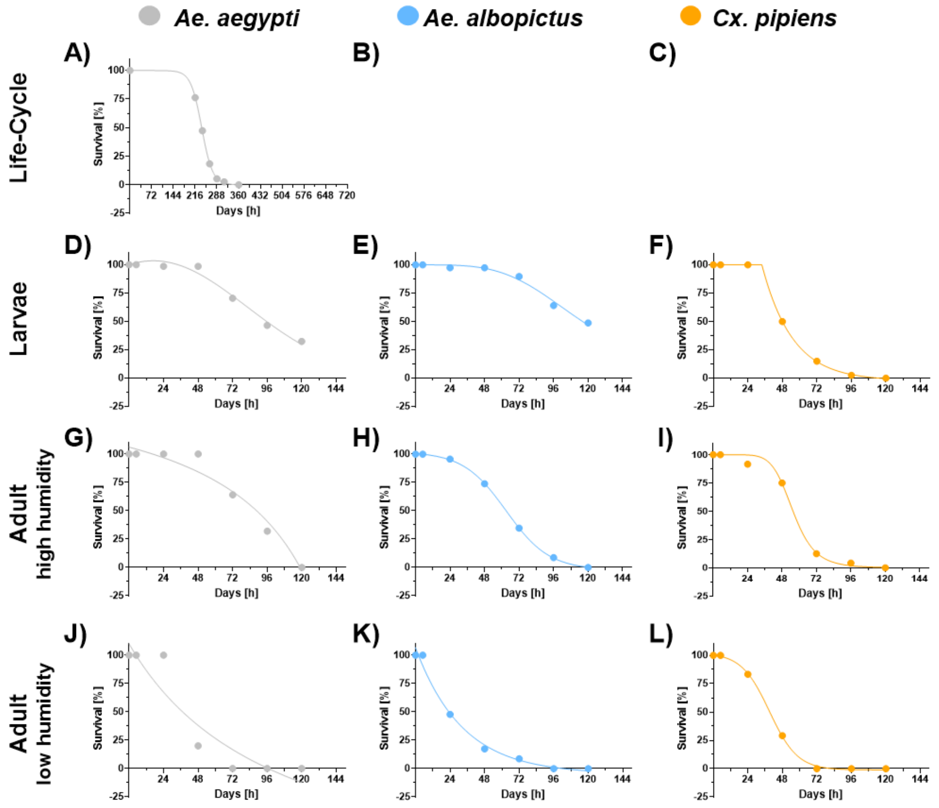

Supplement Figure 13. Non-linear regression models to interpolate upper thermal limits at 36°C. Non-linear regression models were used to interpolate the time point at which 10% of mosquitoes (*Cx. pipiens, Ae. albopictus*, and *Ae. aegypti*) still survived ('days of survival') at 36°C, based on the tested larvae, adult (low and high humidity), and life-cycle experiments. For fitting parameters see Supplement Table 9. Supplement Table 10 shows interpolated days at 10% survival threshold.

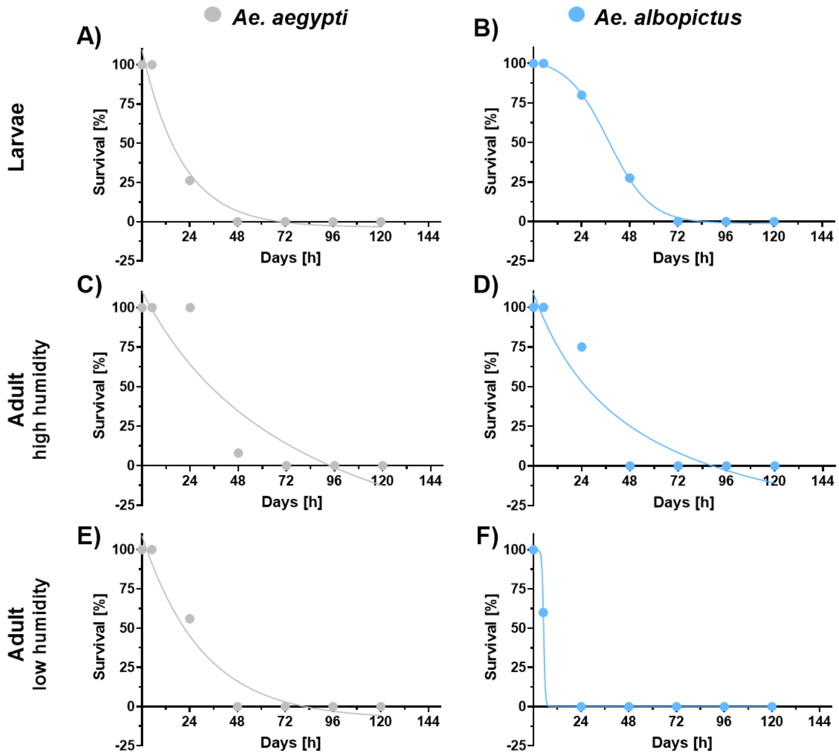

Supplement Figure 14. Non-linear regression models to interpolate upper thermal limits at 40°C. Non-linear regression models were used to interpolate the time point at which 10% of mosquitoes (*Cx. pipiens, Ae. albopictus*, and *Ae. aegypti*) still survived ('days of survival') at 40°C, based on the tested larvae, adult (low and high humidity), and life-cycle experiments. For fitting parameters see Supplement Table 9. Supplement Table 10 shows interpolated days at 10% survival threshold.

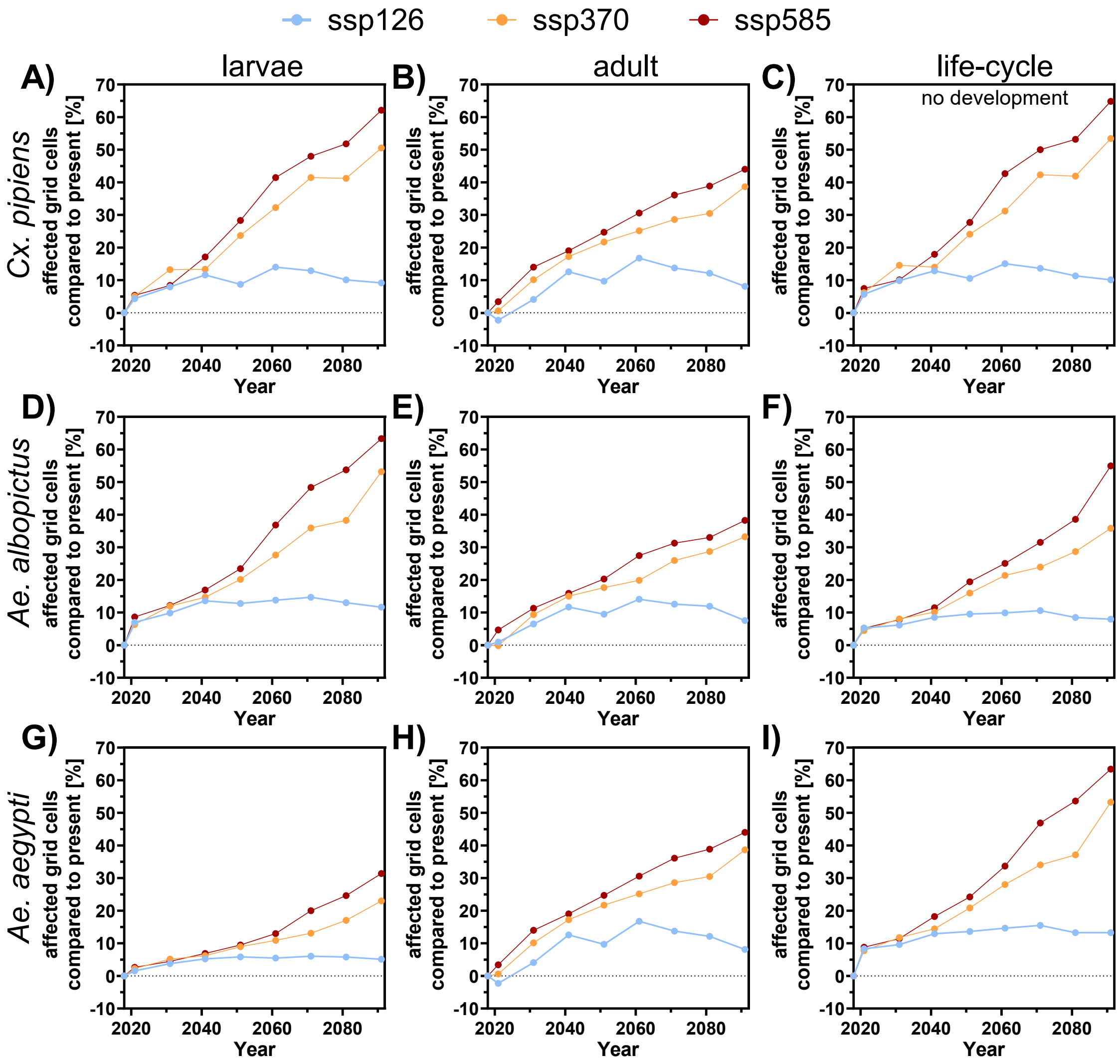

Supplement Figure 15. **Projected increases in the proportion of affected grid cells over time for three mosquito species in Europe at 33 °C.** Shown is the percentage of grid cells experiencing at least one lethal heatwave event per year, relative to the present, under three SSP scenarios (SSP126, SSP370, SSP585; ensemble model). Panels: Species-specific upper thermal limits are for *Cx. pipiens*: larvae >10 days (A), adults >5 days (B), life-cycle >11 days (C); *Ae. albopictus*: larvae >14 days (D) , adults >4 days (E), life-cycle >25 days (F); *Ae. aegypti* larvae: >56 days (G), adults >5 days (H), life-cycle >16 days (I). Changes are analyzed in 10-year intervals from 2021 to 2100. Interpolation details: Supplement Figure 11, 12, 13 and 14, Supplement Table 9.

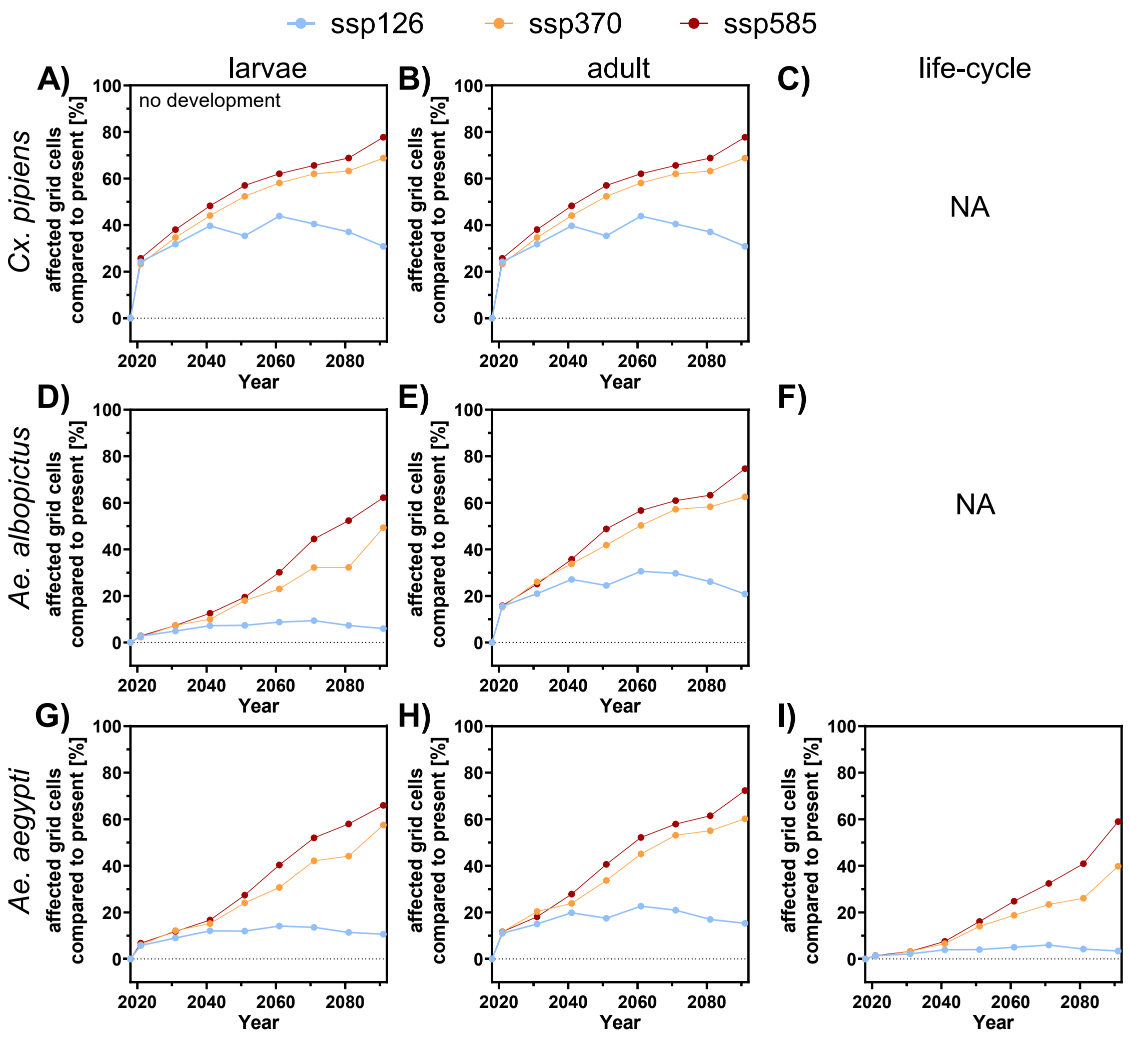

Supplement Figure 16. **Projected increases in the proportion of affected grid cells over time for three mosquito species in Europe at 36 °C.** Shown is the percentage of grid cells experiencing at least one lethal heatwave event per year, relative to the present, under three SSP scenarios (SSP126, SSP370, SSP585; ensemble model). Panels: Species-specific upper thermal limits are for *Cx. pipiens*: larvae >3 days (A), adults >3 days (B), life-cycle >0 days (C); *Ae. albopictus*: larvae >9 days (D) , adults >4 days (E), life-cycle >1 days (F); *Ae. aegypti* larvae: >7 days (G), adults >5 days (H), life-cycle >12 days (I). Changes are analyzed in 10-year intervals from 2021 to 2100. Interpolation details: Supplement Figure 11, 12, 13 and 14, Supplement Table 9.

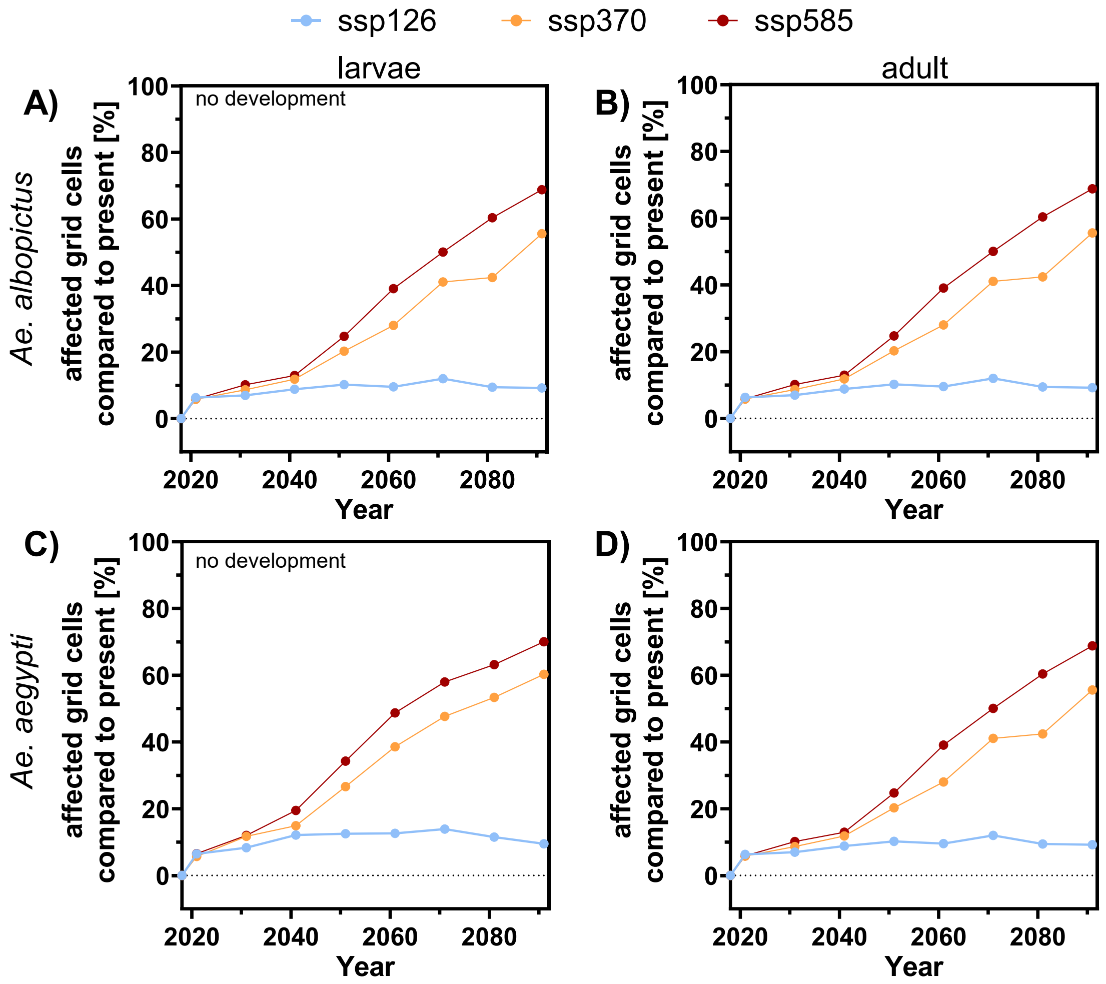

Supplement Figure 17. Projected increases in the proportion of affected grid cells over time for three mosquito species in Europe at 40 °C. Shown is the percentage of grid cells experiencing at least one lethal heatwave event per year, relative to the present, under three SSP scenarios (SSP126, SSP370, SSP585; ensemble model). Panels: A) *Ae. albopictus* larvae (>3 days), B) *Ae. albopictus* adults (>3 days), C) *Ae. aegypti* larvae (>2 days), D) *Ae. aegypti* adults (>3 days). Lethal events are defined as periods when maximum daily temperatures exceed the species-specific upper thermal limits (UTL) given in Table 1. Changes are shown in 10-year intervals from 2021 to 2100, with the present defined as 2014–2024 (dot at 2018). Interpolation methods: Supplementary Figures 11–14 and Supplementary Table 9.
