# Supplementary material for: Heatwaves Constrain the Future Persistence of Mosquito Vectors in Europe
Source: Glob Chang Biol. 2026 Apr 27;32:e70876. doi: 10.1111/gcb.70876 (PMC13112340; doi:10.1111/gcb.70876)
Supplement: Supplementary file 2 — Table S1: Validation of low and high humidity in press‐on‐lid glasses. Test temperature = expected temperature also used in heat experiments. Temperature in the climate cabinet assessed with HOBO data loggers (type MX1104, ONSET). Humidity treatment = High humidity equals 1 mL of water present in the press‐on lid glass, while low humidity equals no water. Temperature = mean temperature measured with a temperature sensor (Thlevel) in press‐on lid glass over 1 h. Humidity = mean humidity measured with a humidity sensor (Thlevel) in press‐on lid glass over 1 h. Thevel loggers were controlled using with HOBO data loggers (type MX1104, ONSET). Table S2: Mean, minimum and maximum temperatures (in °C) tested during heat experiments with eggs, larvae, adults and across the full life‐cycle, including rearing conditions. Temperatures within climate cabinets were tracked using HOBO data loggers (type MX1104, ONSET). Relative humidity in the adult experiment was set to low and high conditions, as described in Table S1, whereas during the life‐cycle experiment, adults were only exposed to high humidity (see Table S1). Table S3: Mean, minimum and maximum temperature (in °C) and relative humidity (in %) tested during heat experiments with eggs. Relative Humidity and temperatures within climate cabinets were tracked using HOBO data loggers (type MX1104, ONSET). Cx. pipiens eggs were tested on water. Aedes eggs were exposed without water. The relative humidity gradually increased over the course of the measurement period, starting from the respective minimum temperatures. Table S4: Amount of adults exposed to 27°C, 30°C, 33°C, 36°C and 40°C for 5 days. Table S5: Fitting parameters of non‐linear regression models for temperature‐dependent survival and development time of mosquito species. Models were used to compare species. Details about raw data compared with the life‐cycle data originating from this study can be found in the literature review in (Kramer 2021). Due to experimental [file GCB-32-e70876-s002.pdf]

Supplementary Tables

Supplement Table 1. Validation of low and high humidity in press on-lid glasses. Test temperature= expected temperature also used in heat experiments. Temperature in the climate cabinet assessed with HOBO data loggers (type MX1104, ONSET ®). Humidity treatment= High humidity equals 1 ml of water present in the press-on lid glass, while low humidity equals no water. Temperature = mean temperature measured with a temperature sensor (Thlevel) in press-on lid glass over 1 hour. Humidity = mean humidity measured with a humidity sensor (Thlevel) in press-on lid glass over 1 hour. Thevel loggers were controlled using with HOBO data loggers (type MX1104, ONSET ®).

Test temperature	temperature environment chamber [°C]	Humidity treatment	temperature in press on lid glass [°C]	humidity in press on lid glass [%]
27°C	26.6	low humidity	27.2	42
	26.7	high humidity	27.2	86
30°C	30.2	low humidity	31.1	38
	30.1	high humidity	31.1	85
33°C	32.6	low humidity	33.3	35
	32.9	high humidity	33.2	84
36°C	35.5	low humidity	36.3	28
	34.9	high humidity	35.8	84
40°C	40.4	low humidity	41.1	27
	40.2	high humidity	41.2	82

Supplement Table 2. **Mean, minimum and maximum temperatures (in °C) tested during heat experiments with eggs, larvae, adults, and across the full life-cycle, including rearing conditions.** Temperatures within climate cabinets were tracked using HOBO data loggers (type MX1104, ONSET ®). Relative humidity in the adult experiment was set to low and high conditions, as described in Supplement Table 1, whereas during the life-cycle experiment, adults were only exposed to high humidity (see Supplement Table 1).

**Experiment**	**Theoretical temperature [°C]**	**Mean±SD [°C]**	**Min. [°C]**	**Max. [°C]**	**Mean±SD [°C]**	**Min. [°C]**	**Max. [°C]**	**Mean±SD [°C]**	**Min. [°C]**	**Max. [°C]**
		Cx. pipiens			***Ae. albopictus***			***Ae. aegypti***		
Larvae	27	26.58±0.33	25.36	27.52	26.58±0.33	25.36	27.52	26.58±0.33	25.36	27.52
	30	30.24±0.21	29.70	30.93						
	33	32.5±0.35	31.86	33.96	32.98±0.55	31.79	35.65	32.54±0.47	31.61	33.90
	36	35.5±0.34	34.72	36.06	36.09±0.51	34.92	37.70	36.11±0.73	34.16	38.34
	40				39.94±0.54	39.03	42.81	40.22±0.49	38.10	41.69
Adults	27	26.52±0.36	25.95	26.52	26.53±0.38	25.75	27.52	26.53±0.38	25.75	27.52
	30	30.34±0.22	29.82	31.24						
	33	32.66±0.29	32.00	33.13	32.62±0.31	31.92	33.22	32.49±0.34	31.56	33.15
	36	35.53±0.32	34.18	36.23	35.76±0.33	34.92	36.25	35.67±0.3	34.76	35.67
	40				39.79±0.31	39.07	40.62	40.1±0.18	38.87	40.63
Behaviour Larvae	27	26.3±0.20	25.36	26.97	26.4±0.15	26.08	26.962	26.4±0.15	26.08	26.96
	33	32.62±0.37	31.96	33.80	33.26±0.19	32.44	33.568	33.26±0.19	32.44	33.57
	36	36.8±0.49	35.61	38.12	36.53±0.12	35.63	36.773	36.53±0.12	35.63	36.77
	40	40.1±0.49	39.03	41.63	40.24±0.28	39.22	40.576	40.24±0.28	39.22	40.58
	Room temperature during experiment	21.69±0.18	21.36	22.09	20.53±0.52	19.43	21.28	21.72±0.33	21.19	22.22
Life-Cycle	27	26.68±0.31	25.95	28.02	26.72±0.30	25.95	28.02	26.70±0.31	25.95	28.02
	30	30.40±0.27	29.69	31.26	30.37±0.28	29.54	31.26			
	33	32.55±0.30	31.90	33.13	32.39±0.34	31.42	33.09	32.24±0.43	31.09	33.28
	36							35.38±0.34	34.34	36.00
Rearing	27	27.12±0.47	25.75	28.01	27.12±0.47	25.75	28.01	27.12±0.47	25.75	28.01

Supplement Table 3. **Mean, minimum and maximum temperature (in °C) and relative humidity (in %) tested during heat experiments with eggs.** Relative Humidity and temperatures within climate cabinets were tracked using HOBO data loggers (type MX1104, ONSET ®). *Cx. pipiens* eggs were tested on water. *Aedes* eggs were exposed without water. The relative humidity gradually increased over the course of the measurement period, starting from the respective minimum temperatures.

**Experiment**	**Theoretical temp. [°C]**	**Humi-dity**	**Mean±SD [°C]**	**Min. [°C]**	**Max. [°C]**	**Mean±SD [%]**	**Min. [%]**	**Max. [%]**	**Mean±SD [°C]**	**Min. [°C]**	**Max. [°C]**	**Mean±SD [%]**	**Min. [%]**	**Max. [%]**	**Mean±SD [°C]**	**Min. [°C]**	**Max. [°C]**	**Mean±SD [%]**	**Min. [%]**	**Max. [%]**
			Ae. albopictus						***Ae. aegypti***						***Cx. pipiens***					
Eggs	27	Low	26.55±0.33	24.64	27.05	48.89±0.59	48.03	53.54	27.23±0.12	26.93	27.37	39.05±1.76	35.01	42.20	27.5±0.2	26.9	27.9	80.9±2.8	51.7	89.7
	27	High	26.54±0.33	26.23	27.05	79.08±8.39	46.40	88.79	27.34±0.19	26.99	27.54	75.09±11.11	38.40	87.75						
	33	Low	33.45±0.53	27.72	34.03	44.18±2.02	41.62	57.74	31.18±0.08	31.00	31.30	37.76±0.60	35.85	38.52						
	33	High	33.28±0.46	28.42	33.79	83.30±8.57	46.70	92.43	31.38±0.08	31.01	31.51	81.25±10.43	40.93	91.55	33.3±0.2	31.1	33.7	89.9±3.2	34.7	92.8
	36	Low	36.07±0.51	26.57	36.48	41.26±1.43	39.28	51.71	33.79±0.10	33.68	34.77	37.71±0.17	37.13	38.47						
	36	High	36.04±0.49	27.24	36.44	84.16±8.67	45.48	92.69	34.38±0.11	34.07	35.35	87.18±8.64	49.99	95.32	36.2±0.3	34.6	36.9	88.7±2.8	37.7	92.8
	40	Low	40.21±0.45	27.44	41.10	42.74±2.46	39.81	63.30	37.90±0.04	37.81	37.96	36.97±0.48	35.99	37.70						
	40	High	40.07±0.50	25.43	40.80	83.88±7.63	56.21	91.44	38.36±0.05	37.97	38.43	84.36±8.57	47.60	92.53	40.3±0.2	36.3	40.6	86.6±4.1	32.7	92.2
Hatching of eggs			27.48±0.20	27.07	28.01				27.26±0.15	26.44	27.89									

Supplement Table 4. Amount of adults exposed to 27°C, 30°C, 33°C, 36°C and 40°C for 5 days.

	**27°C**	**33°C**	**36°C**	**40°C**
		Ae. albopictus		
		High Humidity		
female	7	7	3	4
male	15	18	20	16
		Low Humidity		
female	8	7	3	1
male	14	18	20	19
		Ae. aegypti		
		High Humidity		
female	5	5	5	4
male	20	20	20	21
		Low Humidity		
female	3	6	2	3
male	22	19	23	22
	**27°C**	**30°C**	**33°C**	**36°C**
		Cx. pipiens		
		High Humidity		
female	6	7	11	10
male	19	18	14	14
		Low Humidity		
female	7	10	8	12
male	18	15	17	12

Supplement Table 5. Fitting parameters of non-linear regression models for temperature-dependent survival and development time of mosquito species. Models were used to compare species. Details about raw data compared with the life-cycle data originating from this study can be found in the literature review in (Kramer, 2021). Due to experimental differences within the studies used for data extraction - such as variations in larval feeding - outliers were removed from the full dataset prior to performing non-linear regression analysis (Q=1%). Survival (%) and development (in days) from L1-adult emergence were analyzed worldwide for all populations were literature data was extracted from (Kramer, 2021; Moser et al., 2023), as well as for the survival of the tested populations of this study (*Ae. aegypti* – Nepal, *Ae. albopictus* – Italy, *Cx. pipiens* – Belgium).

	**Survival (data from literature review)**			**Development (data from literature review)**			**Survival (populations tested)**		
**Species**	*Ae. aegypti*	*Ae. albopictus*	*Cx. pipiens*	*Ae. aegypti*	*Ae. albopictus*	*Cx. pipiens*	*Ae. aegypti*	*Ae. albopictus*	*Cx. pipiens*
**Model**	Lognormal			Second order polynomial			Centered second order polynomial (quadratic)		
**p-value**	<0.0001			<0.0001			<0.0001		
**Outliers removed**	2	0	0	5	0	6	NA	NA	NA
**R^2**	0.72	0.79	0.53	0.90	0.91	0.58	0.83	0.86	0.72
**Sy.x**	19.64	18.29	20.19	1.7	2.2	3.8	15.34	16.67	19.53

Supplement Table 6. Significance level of Kaplan-Meier survival curves for heat exposure experiments over the 5-days lasting experimental exposure. A) Larvae (L) of *Cx. pipiens*, *Ae. albopictus* and *Ae. aegypti* were exposed to 27°C, 33°C, 36°C and 40°C for 5d, *Cx. pipiens* larvae were also tested at 30°C. B) Adults of *Cx. pipiens*, *Ae. albopictus* and *Ae. aegypti* were exposed to 27°C, 33°C, 36°C and 40°C for 5d at low humidity (A), *Cx. pipiens* larvae were also exposed to 30°C but not to 40°C. C) Adults of *Cx. pipiens*, *Ae. albopictus* and *Ae. aegypti* were exposed to 27°C, 33°C, 36°C and 40°C for 5d at high humidity (A-H), *Cx. pipiens* larvae were also exposed to 30°C but not to 40°C. D) Adult: Comparison of the high and low humidity treatment and temperatures described in B,C. E) Adult vs. larvae: Comparison of the adult compared to the larvae survival described in A, B and C. A-C) p-values are given for *Aedes* in italic and for *Cx. pipiens* in regular. Adjusted p-values are reported when more than two groups are compared (test: Holm-Šídák). ns = not signifiant, NA = not available because it was not tested. Icons used were created with BioRender.com.

A)

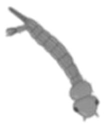

	**27°C**	**30°C***	**33°C**	**36°C**	**40°C**
**27°C**			ns	*<0.05*	*<0.05*
**30°C***	ns		*<0.05*	*<0.05*	*<0.05*
**33°C**	<0.05	<0.05		*<0.05*	*<0.05*
**36°C**	<0.05	<0.05	<0.05		*<0.05*
**40°C**	<0.05	<0.05	<0.05	<0.05	

between species

	Ae. aegypti	***Ae. albopictus***	***Cx. pipiens***
**27°C**		ns	
**33°C**		< 0.05 except *Ae. aegypti* vs. *Ae. albopictus*	
**36°C**		<0.05	
**40°C**		<0.05	

B)

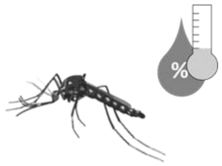

	**27°C**	**30°C***	**33°C**	**36°C**	**40°C**
**27°C**			*<0.05*	*<0.05*	*<0.05*
**30°C***	ns		*<0.05*	*<0.05*	*<0.05*
**33°C**	<0.05	ns		*ns*	*<0.05*
**36°C**	<0.05	ns	<0.05		*<0.05*
**40°C**	<0.05	<0.05	<0.05		

between species

	Ae. aegypti	***Ae. albopictus***	***Cx. pipiens***
**27°C**	0.05 others ns		
**33°C**		ns	
**36°C**		ns	
**40°C**	<0.05		

C)

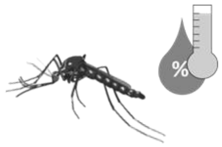

	**27°C**	**30°C***	**33°C**	**36°C**	**40°C**
**27°C**			*<0.05*	*<0.05*	*<0.05*
**30°C***	ns		*<0.05*	*<0.05*	*<0.05*
**33°C**	<0.05	<0.05		*ns*	*<0.05*
**36°C**	<0.05	<0.05	ns		*<0.05*
**40°C**	<0.05	<0.05			

between species

	Ae. aegypti	***Ae. albopictus***	***Cx. pipiens***
**27°C**		ns	
**33°C**		ns	
**36°C**		<0.05, except *Ae. albopictus* vs *Cx. pipiens*	
**40°C**	<0.05		na

D)

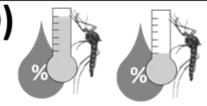

	Ae. aegypti	***Ae. albopictus***	***Cx. pipiens***
**27°C**	ns	<0.05	<0.05
**33°C**	<0.05	<0.05	ns
**36°C**	<0.05	<0.05	<0.05
**40°C**	<0.05	<0.05	na

E)

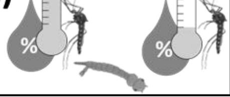

	Ae. aegypti	***Ae. albopictus***	***Cx. pipiens***
**27°C**	<0.05 except A vs A-H	<0.05	<0.05
**33°C**	<0.05	<0.05	<0.05, except A vs A-H
**36°C**	<0.05	<0.05	<0.05, except L vs A-H
**40°C**	<0.05	<0.05	na

*30°C was only tested in *Cx. pipiens*

Supplement Table 7. **Life-history parameters analysed after temperature exposure to 27°C, 30°C, 33°C or 36°C of *Cx. pipiens*, *Ae. albopictus* and *Ae. aegypti* from L1 until death.** ELS = entire life span (days) in brackets number of individuals that survived until adult stage. L1-adult = aquatic life span (days) in brackets individuals survived until emergence. Survival = survival of individuals until emergence compared to larvae still alive after 48h. Sex ratio = male mosquitoes/sum of adult mosquitoes.

Species	Parameter	27°C	30°C	33°C	36°C
Cx. pipiens		18.4±2.8 (19)	14.0±2.5 (9)	0.0	NA
Ae. albopictus	ELS	20.1±2.6 (38)	15.0±1.9 (38)	20.4±5.5 (5)	NA
Ae. aegypti		17.8 ±2.4 (34)	NA	13.8±2.1 (36)	11.5±1.2 (17)
Cx. pipiens		12.9±2.0 (21)	11.0±1.2 (13)	0.0	NA
Ae. albopictus	L1-adult	13.3±1.7 (38)	10.4±1.2 (38)	16.7±4.7 (6)	NA
Ae. aegypti		11.6±1.1 (34)	10.1±0.8 (36)	NA	9.6±0.6 (19)
Cx. pipiens		54.3±17.9 (35)	23.6±16.2 (38)	0.0 (35)	NA
Ae. albopictus	Survival	95.0±6.8 (42)	95.0±6.8 (40)	13.2±10.1 (38)	NA
Ae. aegypti		91.4±7.7 (38)	92.5±6.85 (40)	NA	43.2±13.2 (39)
Cx. pipiens		0.71	0.62	0.00	NA
Ae. albopictus	Sex ratio	0.49	0.71	0.86	NA
Ae. aegypti		0.47	NA	0.51	0.40

Supplement Table 8. **Comparison of extreme heat exposure results (=maximum exposure duration tested i.e. mean survival after 5 days) of larvae vs. adult (high and low humidity).** Groups were compared using Mann-Whitney tests including multiple comparison tests using the Holm-Šídák method to correct p-values (alpha: 0.05). Adjusted p-values are given.

Temperature	Experiment	*Ae. aegypti*	*Ae. albopictus*	*Cx. pipiens*
	larvae vs. adults low humidity	0.0005	0.0005	0.0004
27°C	larvae vs. adults high humidity	0.0279	0.0021	ns
	adults high humidity vs. adult low humidity	ns	ns	ns
	larvae vs. adults low humidity	NA	NA	<0.0001
30°C	larvae vs. adults high humidity	NA	NA	0.0009
	adults high humidity vs. adult low humidity	NA	NA	ns
	larvae vs. adults low humidity	<0.0001	<0.0001	<0.0001
33°C	larvae vs. adults high humidity	0.0001	<0.0001	<0.0001
	adults high humidity vs. adult low humidity	ns	ns	ns
	larvae vs. adults low humidity	<0.0001	<0.0001	ns
36°C	larvae vs. adults high humidity	<0.0001	<0.0001	ns
	adults high humidity vs. adult low humidity	ns	ns	ns
	larvae vs. adults low humidity	ns	<0.0001	NA
40°C	larvae vs. adults high humidity	<0.0001	ns	NA
	adults high humidity vs. adult low humidity	0.0002	<0.0001	NA

Supplement Table 9. **Fitting parameters of non-linear regression models to interpolate upper thermal limits.** Model and fitting parameters for best-fitted curves using non-linear regression to interpolate unknowns from standard curves within the heat exposure experiment, covering the full life cycle, larvae, and adults under low and high humidity conditions tested at 27°C, 33°C, 36°C, and 40°C (see Table 1, Supplement Figure 11, 12, 13 and 14).

Temperature	Model description	*Ae. aegypti*	*Ae. albopictus*	*Cx. pipiens*
		**Life Cycle**		
**27°C**	model	Sigmoidal, 4PL, X is concentration	Asymmetric Sigmoidal, 5PL, X is concentration	Plateau followed by one phase decay
	R square	0.9955	0.9979	0.9892
	sum of square	85.15	38.57	190.8
	Sy.x	3.076	2.07	3.831
**33°C**	model	Plateau followed by one phase decay	[Inhibitor] vs. normalized response -- Variable slope	Pade (1,1) approximant
	R square	0.9989	0.9908	0.9839
	sum of square	17.42	194.1	89.44
	Sy.x	1.578	3.379	9.457
**36°C**	model	log(inhibitor) vs. normalized response -- Variable slope		
	R square	0.9998	Not tested	Not tested
	sum of square	2.19		
	Sy.x	0.6619		
		**Larvae**		
**27°C**	model	Line	Linear quadratic survival (Y is percentage)	Linear quadratic survival (Y is percentage)
	R square	1	0.8995	0.97
	sum of square	0	14.16	1.5
	Sy.x		1.683	0.5477
**33°C**	model	Line	[Inhibitor] vs. norm. resp.- Var.slo.	Line
	R square	0.9533	0.9977	0.8836
	sum of square	2.837	0.4543	247.1
	Sy.x	0.7533	0.3014	7.03
**36°C**	model	Linear quadratic survival (Y is percentage)	[Inhibitor] vs. normalized response -- Variable slope	Plateau followed by one phase decay
	R square	0.9836	0.9871	0.9999
	sum of square	81.1	32.74	0.8638
	Sy.x	4.027	2.559	0.5366
**40°C**	model	Dissociation - One phase exponential decay	Sigmoidal, 4PL, X is log(concentration)	
	R square	0.9693	0.9992	
	sum of square	411.1	10.91	
	Sy.x	10.14	1.907	died
		**Adult - low humidity**		
**27°C**	model	Sigmoidal, 4PL, X is log(concentration)	Plateau followed by one phase decay	[Inhibitor] vs. normalized response -- Variable slope
	R square	0.9999	0.9995	0.9871
	sum of square	0.5611	5.966	110.5
	Sy.x	0.4325	1.41	4.701
**33°C**	model	Pade (1,1) approximant	[Inhibitor] vs. normalized response -- Variable slope	Sigmoidal, 4PL, X is concentration
	R square	0.878	0.9993	0.9998
	sum of square	1924	9.282	2.288
	Sy.x	21.93	1.363	0.8733
**36°C**	model	Pade (1,1) approximant	Dissociation - One phase exponential decay	Sigmoidal, 4PL, X is log(concentration)
	R square	0.878	0.9877	0.9993
	sum of square	1924	147.4	9.029
	Sy.x	21.93	6.07	1.735
**40°C**	model	Dissociation - One phase exponential decay	log(inhibitor) vs. normalized response	
	R square	0.9574	1	not tested
	sum of square	586.5	4.444E-07	
	Sy.x	12.11	0.0002722	
		**Adult - high humidity**		
**27°C**	model	Second order polynomial (quadratic)	log(inhibitor) vs. normalized response -- Variable slope	[Inhibitor] vs. normalized response -- Variable slope

	R square	0.9802	0.9831	0.9943
	sum of square	76.35	111.8	20.68
	Sy.x	4.369	4.73	2.034
**33°C**	model	Pade (1,1) approximant	Sigmoidal, 4PL, X is log(concentrat ion)	Line
	R square	0.9828	0.999	0.9678
	sum of square	125.8	10.4	345.5
	Sy.x	5.609	1.862	8.313
**36°C**	model	Pade (1,1) approximant	Sigmoidal, 4PL, X is log(concentrat ion)	[Inhibitor] vs. normalized respon se -- Variable slope
	R square	0.9594	0.9999	0.9945
	sum of square	405.4	1.091	72.13
	Sy.x	10.07	0.6029	3.798
**40°C**	model	Pade (1,1) approximant	Pade (1,1) approximant	
	R square	0.8511	0.9032	
	sum of square	2459	1434	
	Sy.x	24.79	18.93	not tested

Supplement Table 10. **Species-specific interpolated days until survival dropped below 10% at >30°C.** For the egg stage, only *Aedes* species with survival rates above 10% are shown, since *Culex pipiens* eggs either hatched during the exposure period or failed to survive. For adults low and high humidity are given but for maps and future predictions only the high humidity treatment was used. Details in Supplement Table 9 and Supplement Figure 11, 12, 13 and 14). Whether development occurred was indicated based on Supplementary Figures 7 and 8.

ID	Population origin	Species	Lifestage	Temperature	Level humidity high/low	Survivial [in days]	Development occurs
1	Nepal, Kathmandu	*Aedes aegypti*	larvae	**33**	na	56	Yes
2			adult	**33**	low	3	-
3			adult	**33**	high	5	-
4			Life-cycle	**33**	na	16	Yes
5			eggs	**36**	high	10	-
6			larvae	**36**	na	7	Yes
7			adult	**36**	low	3	-
8			adult	**36**	high	5	-
9			Life-cycle	**36**	na	12	Yes
10			larvae	**40**	na	2	No
11			adult	**40**	low	2	-
12			adult	**40**	high	3	-
13	Italy, Terni	*Aedes albopictus*	eggs	**33**	low	10	-
14			larvae	**33**	na	14	Yes
15			adult	**33**	low	2	-
16			adult	**33**	high	4	-
17			Life-cycle	**33**	na	25	Yes
18			larvae	**36**	na	9	Yes
19			adult	**36**	low	3	-
20			adult	**36**	high	4	-
21			larvae	**40**	na	3	No
22			adult	**40**	low	0	-
23			adult	**40**	high	3	-
24	Belgium, Antwerp	*Culex pipiens molestus*	larvae	**33**	na	10	Yes
25			adult	**33**	low	3	-
26			adult	**33**	high	5	-
27			Life-cycle	**33**	na	11	No
28			larvae	**36**	na	3	No
29			adult	**36**	low	3	-
30			adult	**36**	high	3	-
31			larvae	**40**	na	0	No
